# Simultaneous Production of Rhamnolipids and Glycolipopeptides by *Burkholderia thailandensis* E264 via Covalorization of Torrefied Wood Waste and Food Waste

**DOI:** 10.1021/acsomega.5c11012

**Published:** 2026-01-12

**Authors:** Anjana Hari, Ernesto Zapata, Michaela Hříbková, Koit Herodes, Vahur Rooni, Sabarathinam Shanmugam, Cristiana A. V. Torres, Timo Kikas

**Affiliations:** † Chair of Biosystems Engineering, Institute of Forestry and Engineering, 85334 Estonian University of Life Sciences, Kreutzwaldi 56, Tartu 51014, Estonia; ‡ Institute of Chemistry, 37546 University of Tartu, Ravila 14a, 50411 Tartu, Estonia; § i4HB - Institute for Health and Bioeconomy, NOVA School of Science and Technology, 119482 NOVA University Lisbon, Caparica 2829-516, Portugal; ∥ UCIBIO − Applied Molecular Biosciences Unit, Department of Chemistry, NOVA School of Science and Technology, NOVA University Lisbon, Caparica 2829-516, Portugal

## Abstract

Continued research into structurally and functionally diverse biosurfactants is crucial to identify new biosurfactants that are cost-competitive and suitable for diverse industrial niches. Herein, we report the successful application of wood waste torrefied at 225 °C (T225) and food waste (FWS) as media components in three different formulations—T225, T225–FWS, and FWS—for the concomitant production of rhamnolipids and biopolymers by *Burkholderia thailandensis* E264. The rhamnolipids (carbon chain length C8–C16) exhibited media-dependent congener differences, and the biopolymers were identified to be glycolipopeptides with molecular weights of 4.4 × 10^5^–5.5 × 10^5^ Da. T225–FWS exhibited the highest thermostability and retained ∼30% of its residual mass at 400 °C, suggesting potential high-temperature applications, including oil recovery and industrial cleaning. Rhamnose was the dominant sugar in T225 and T225–FWS glycolipopeptides, whereas in FWS, galactose was dominant. FAME analysis by GC–MS revealed lipid chains ranging from C12 to C18 in the biopolymers. All three biopolymers were anionic and possessed β-hemolytic, emulsification, and drop-collapse activities against crude oil and engine oil. Moreover, T225, T225–FWS, and FWS biopolymers could reduce the surface tension of water up to 36.8, 35.4, and 48.5 mN/m, respectively. This study could lead to resource-efficient synthesis of structurally and, hence, functionally tunable biosurfactants from low-cost, completely waste-derived media in biorefinery operations.

## Graphical abstract

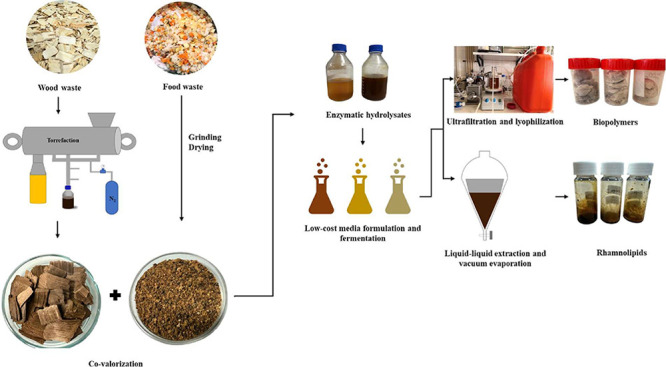

## Introduction

Chemical industries are responsible for the annual emission of approximately 2 billion metric tons of CO_2_ worldwide.^1^ Achieving decarbonization of this industry is crucial to achieving net-zero emissions. However, this is particularly challenging because of the industry’s energy- and CO_2_-intensive nature in terms of process energy as well as carbon-rich, fossil fuel-based feedstock, which is distributed across several supply chains.^1,2^ Biomass is an alternative source of carbon and hydrogen atoms for chemical synthesis.^3^ Replacing fossil fuel-based feedstock with sustainable biomass-based feedstock is a step toward achieving net-zero emissions in the chemical industry.

One such example is the case of surfactants. Surfactants are compounds of amphiphilic nature that stabilize the interface between two immiscible phases (such as oil and water) by orienting their hydrophobic tails toward the nonpolar phase and their hydrophilic heads toward the polar phase, thereby reducing interfacial tension. This property has made them an indispensable component of our daily life, as an ingredient in daily use personal care formulations, cleaning agents, drugs and pharmaceuticals, pesticides, dispersants, emulsifiers, and wetting agents.^4^ Synthetic surfactants are currently synthesized from oil and natural gas, which is refined to form petrochemicals, such as ethane and propane, which are then transformed by a high-temperature cracking process to form intermediates, such as ethylenes and benzenes. Subsequently, functional groups are added via acid catalysis to yield synthetic surfactants.^5,6^ The wide spectrum of applications of such synthetic surfactants, along with the long duration of time required for these compounds to be completely degraded result in their accumulation in the environment, leading to environmental toxicity.^7^ Thus, despite their low cost and high efficiency, there is a pressing need and associated rise in demand for alternatives, such as microbial biosurfactants.

Microbial biosurfactants are reported to have improved biodegradability and biocompatibility as well as low toxicity.^8^ They can be classified based on their molecular weight as low- (<1200 Da) and high-molecular-weight biosurfactants (>45,000 Da) or biopolymers.^9^ They are further classified based on the composition of the hydrophilic and hydrophobic moieties as glycolipids, fatty acids, phospholipids and neutral lipids, lipopeptides, polymeric biosurfactants, and particulate biosurfactants.^10^ Rhamnolipids, a type of glycolipid composed of l-(+)-rhamnose and β-hydroxyalkanoic acid units, are among the most studied group. Their ability to reduce surface tension and stabilize emulsions as well as various biological activities have supported their application in various fields such as cleaning agents, cosmetics, pharmaceuticals, oil recovery, agriculture, and bioremediation.^11^ Moreover, rhamnolipids have been commercialized by various companies such as Evonik Industries, AGAE Technologies, Stepan Company, and Jeneil Biosurfactant.^12^ Despite this progress, rhamnolipids are not completely ideal due to multiple limitations including excessive foaming and regulatory constraints due to their pathogenic origin. Further, process and raw material costs limit the economics of the rhamnolipid production process, demonstrating the need for cost reduction.^13^ Therefore, there is a need to explore other structurally diverse biosurfactant molecules that can complement rhamnolipids in safety, performance, and fine-tuned functionality for specific applications. It would be advantageous if such biosurfactants could be produced using media other than pure sugars, such as those derived from widely available lignocellulosic waste-streams. Such an approach could balance process costs while executing appropriate resource recovery from waste.^14^ Estonia generates a lot of unprocessed or minimally processed wood waste—lignocellulosic waste derived from forestry and associated industries—which is currently used for district heating as there are few alternative high-value valorization routes available.^15^ Moreover, food waste leading to an economic loss of 164 million euros is generated annually in Estonia, which also has a considerable environmental impact.^16^ These waste-streams could contribute toward the production of high-value products from low-value waste, leading to optimal resource recovery. Moreover, differences in media composition have been known to influence biosurfactant composition and structure, which would even allow structure optimization for different applications.^17^

However, a known challenge in the use of such complex waste-streams is the heterogeneity of the substrate, which complicates downstream processing steps. Hence, it is also necessary to pretreat the waste-streams to confer homogeneity and break down the complex compounds into simpler molecules that can be readily utilized by the biosurfactant-producing microorganism.^18^ We have previously demonstrated a proof-of-concept process of biosurfactant production using *Burkholderia thailandensis* E264, a rhamnolipid-producing, nonpathogenic (BioSafety Level 2) microorganism that utilized wood waste pretreated by torrefaction.^19^ The crude biosurfactant produced via the process had exceptional emulsion stability as well as surface and interfacial tension-lowering activity; however, it had low yield. Thus, in this study, we aim to study the chemical composition of the biosurfactant derived from torrefied wood waste hydrolysate, which could be responsible for its surface activity.

Torrefaction is a mild thermochemical pretreatment using temperatures of 200–300 °C at atmospheric pressure in the absence of oxygen for short residence times (up to 1 h). It requires lesser energy input than pretreatments using pressure, specialized chemicals, and much higher temperature (such as pyrolysis for bio-oil generation, which operates in the range of 400–550 °C).^20^ Additionally, with the right reactor configurations for torrefaction, energy can be recovered via the combustion of volatiles released during torrefaction, allowing for autothermal operation based on other factors such as biomass moisture content, temperature, inert gas requirement, etc.^21–24^ Torrefied biomass or biocoal, the major product of torrefaction at 300 °C, has a market price of approximately USD 105 per ton; although platform chemicals such as furfural and 5-hydroxy methyl furfural are also produced, their yields are traditionally very low.^19^ Thus, exploring torrefied biomass as a substrate for higher value bioproducts, such as biosurfactants, is beneficial.

In addition, to explore strategies to improve the process while reducing raw material costs, we present the covalorization of torrefied wood waste with food waste, with the hypothesis that food waste can provide extraneous lipid precursors for *B. thailandensis* E264 to produce novel biosurfactant compounds in addition to rhamnolipids. To the best of our knowledge, this is the first study to report the simultaneous production of rhamnolipids and high-molecular-weight polymeric biosurfactants or biopolymers by *B. thailandensis* E264 from three different waste-derived media. With this strategy, we describe process improvement at different levels: (i) the use of a nonpathogenic species that is able to grow on different complex waste-streams;^25,25–27^ (ii) improved resource recovery from widely available wood and food waste; and (iii) the possibility of obtaining diverse structures of biosurfactants using tailored media compositions.

## Materials and Methods

### Biomass Collection and Torrefaction

Aspen wood chips were obtained from the AS Estonian Cell, Lääne-Virumaa, Estonia. These were air-dried for 2 weeks indoors before torrefaction. Torrefaction of wood waste was performed in an inert N_2_ atmosphere at 225 °C for 1 h in a continuous torrefaction reactor system as described in Cahyanti et al.^21^ The reactor was heated with ceramic heaters. The heater temperatures were controlled using E6500 proportional-integral-derivative controllers (Evikon MCI OÜ, Tartu, Estonia). The temperature data was recorded continuously using a eight-channel TC-08 Thermocouple data logger (Pico technology Ltd., Cambridgeshire, United Kingdom). The biomass residence time inside the reactor was controlled by an intelligent vector control micro drive VFD-C200 with a built-in programmable logic controller function (Delta electronics Inc., Tainan, Taiwan) by adjusting the frequency. In order to maintain an inert environment inside the reaction chamber and to move volatile compounds, nitrogen gas was constantly flushed through the reactor. Nitrogen flow rate was controlled using a flow meter (Vögtlin Instruments GmbH, Herne, Germany). Initially, the reactor was flushed with nitrogen at a rate of 10 L/min for 10 min. Later, nitrogen flow was reduced to 5 L/min throughout the experiment. The torrefied wood chips were cooled, collected, milled to <4 mm size using a Retsch SM100 mill (Retsch GmbH, Haan, Germany), and stored until further experiments. The proximate and ultimate characteristics of torrefied wood waste are mentioned in our previous publication.^19^

Food waste was collected daily for 2 weeks from a local kindergarten. It was then ground in a meat grinder (Koneteollisuus OY, Klaukkala, Finland) and oven-dried at 80 °C. The dried food waste was stored in clean plastic boxes at room temperature until further use.

### Enzymatic Hydrolysis

Briefly, 5 g of torrefied wood waste and 5 g of food waste were taken separately in 500 mL screw-capped bottles, to which 1.5 mL of the enzyme MetZyme SUNO 400 (LOT-23–0051; MetGen OY, Kaarina, Finland) and 98.5 mL of citrate buffer (pH 4.8) were added to make up the final volume of 100 mL. MetZyme SUNO is a proprietary blend for lignocellulosic sugars, and it was selected as it gave the best results in the hydrolysis of recalcitrant torrefied wood waste based on the release of fermentable sugars in preliminary experiments. The reactions were incubated in an orbital shaker at 50 °C for 48 h at 220 rpm. After incubation, the hydrolysates were collected by centrifugation at 8000 rpm for 10 min.

### Bacterial Culture

Lyophilized culture of *Burkholderia thailandensis* E264 (DSM 13276) was obtained from the Leibniz Institute DSMZ-German Collection of Microorganisms and Cell Cultures. The bacteria were revived and maintained in nutrient broth or agar according to the DSMZ protocol. For long-term storage at – 20 °C, 30% glycerol stocks were used. Before experiments, the culture was reactivated by suspending a loopful of the glycerol stock in 5 mL of nutrient broth and incubating the mixture in an orbital shaker at 28 °C overnight.

### Media Formulation and Biosurfactant Production

Fermentation media were formulated by mixing the components and making up to a final volume of 200 mL using Bushnell–Haas salts (magnesium sulfate 0.2 g/L, calcium chloride anhydrous 0.02 g/L, potassium dihydrogen phosphate 1 g/L, dipotassium hydrogen phosphate 1 g/L, ammonium nitrate 1 g/L, ferric chloride 0.05 g/L; HiMedia Laboratories Private Ltd., Maharashtra, India) and wood and/or food hydrolysates as shown in Table 1. The pH of the medium was adjusted to 7 using 2 M NaOH.

**Table 1:** Fermentation Media Composition

				macromolecular nutrient composition of the medium (based on enzymatic hydrolysate derived from 5 g of each waste sample)		
medium	Bushnell–Haas (mL)	wood hydrolysate (mL)	food hydrolysate (mL)	total sugars (g/200 mL)	total lipids (g/200 mL)	total protein (g/200 mL)
T225	100	100		4.6	0.08	0.01
FWS	100		100	46.9	0.26	0.03
T225–FWS	100	50	50	25.7	0.17	0.02

For biosurfactant production, 200 mL shake-flask experiments were set up in 500 mL flasks with 2% inoculum concentration. The flasks were incubated at 28 °C and 200 rpm for 6 days. After incubation, the cultures were harvested by centrifugation at 12,000 rpm for 15 min. The supernatants were collected and stored at 4 °C until further processing.

### Rhamnolipid Extraction

For rhamnolipid extraction, the protocol by Funston et al.^28^ was followed with slight modifications. The supernatants were acidified to pH 2 using concentrated HCl, followed by extraction with ethyl acetate thrice. Then, 0.3 g of anhydrous MgSO_4_ per 100 mL of ethyl acetate was added to the organic fractions to remove aqueous traces, followed by filtration and rotary vacuum evaporation. The crude extracts were weighed before analyses.

### Biopolymer Concentration

To recover biopolymers, the remaining supernatant was concentrated using diafiltration/ultrafiltration in a crossflow module (Sartocon Slide Holder, Sartorius, Göttingen, Germany) using a 30 kDa membrane with a surface area of 0.1 m^2^ (HydroSart, Sartorius, Göttingen, Germany), according to Gil et al.^29^ To remove low-molecular-weight compounds, the cross-flow module was operated in diafiltration mode against deionized water, until the conductivity of the retentate was below 10 μS cm^–1^. Afterward, the retentate was concentrated by ultrafiltration and lyophilized (ScanVac CoolSafe, LaboGene, Lillero̷d, Denmark), weighed, and stored in airtight vessels for further analyses. Duplicate samples were used for the studies.

### Composition Analysis of Rhamnolipids and Biopolymers

#### Rhamnolipid Characterization

As rhamnolipids are already well-characterized compounds, Fourier transform infrared (FTIR)–attenuated total reflectance (ATR) spectroscopy and liquid chromatography–mass spectrometry (LC-MS) were used to confirm their presence in the crude extracts. The FTIR-ATR spectra of the crude extracts were measured in the region of 4500–500 cm^–1^using a PerkinElmer Spectrum Two FT-IR spectrometer (PerkinElmer Inc., Massachusetts, USA) equipped with a lithium tantalate (LiTaO_3_) detector at a resolution of 0.5 cm^–1^ and 8 scans.

For LC-MS/MS analyses, the crude extracts were dissolved in 10 mL of methanol, further diluted with methanol in a 1:4 ratio, and filtered (regenerated cellulose syringe filters 0.2 μm pore size; Phenomenex Inc., California, USA) before injection. Chromatographic separation was carried out in a Shimadzu Nexera X2 UHPLC system (Shimadzu Scientific Instruments, Inc., Maryland, USA) coupled to a Shimadzu LCMS-8050 (Shimadzu Scientific Instruments, Inc., Maryland, USA) triple quadrupole mass spectrometer with an electrospray ionization source (ESI). A Zorbax Eclipse Plus C18 column (50 × 2.1 mm; 1.8 μm; Agilent Technologies, California, USA) was used. The mobile phases used were the following: A, 0.1% formic acid in water, and B, acetonitrile. The gradient elution program used was as follows: (% of component B is given): 0–2 min, 40%; 2–20 min, 40–95%; 20–24 min, 95%; 24–26 min, 95–40%; and 26–30 min, 40%. The column temperature was 40 °C, the flow rate was 0.3 mL/min, and the injection volume was 2 μL. For ESI, the following parameters were used: nebulizer gas flow: 3 L/min, heating gas flow at 10 L/min, drying gas flow at 10 L/min, interface temperature: 300 °C, desolvation line temperature: 250 °C, and heat block temperature: 400 °C. A flash-chromatography-purified extract of *B. thailandensis* rhamnolipids grown on 4% (w/v) glycerol was used as a standard. Instrument control and data acquisition were performed using Shimadzu LabSolutions Software (Shimadzu Corporation, Kyoto, Japan). For MS detection, full scans and product ion scans were done in positive and negative ionization modes. Marvin was used for drawing chemical structures, substructures, and reactions (Marvin version 25.3.0).^30^

#### Biopolymer Characterization

##### Elemental Analysis

Elemental analysis was performed for the lyophilized samples using an elemental analyzer (Thermo Finnigan-CE Instruments, Flash EA 1112 CHNS series, Milan, Italy). The lyophilized samples were subjected to flash combustion in an oxygen environment, and the resulting gases (N_2_, CO_2_, H_2_O, and SO_2_) were separated by gas chromatography. Finally, the content of nitrogen, carbon, hydrogen, and sulfur was calculated using a thermal conductivity detector, and the results were analyzed using the preinstalled software Eager 300 (Thermo Fisher Scientific GmbH, Bremen, Germany). The analyses were run on duplicate samples, and the results are expressed as average ± standard deviation. The oxygen content (%) in the samples was calculated using the following formula:

O=100−(C+H+N+S) 1

where O, C, H, N, and S are the percentage contents of oxygen, carbon, hydrogen, nitrogen, and sulfur in each sample.

##### FTIR

The FTIR-ATR spectra of the lyophilized samples were measured in the region of 4500–500 cm^–1^ using a PerkinElmer Spectrum Two FT-IR spectrometer (PerkinElmer Inc., Massachusetts, USA) as described in the previous section.

##### Proton Nuclear Magnetic Resonance (^1^H NMR) Spectroscopy

Samples were dissolved in d6-dimethyl sulfoxide (d6-DMSO) and filtered through cotton wool for NMR analysis. ^1^H NMR spectra were acquired using a Bruker Avance-III 700 MHz NMR spectrometer equipped with a 5 mm triple broadband inverse (TBI) probe (Bruker Corporation, Fällanden, Switzerland) at 25 °C. The acquired spectra were analyzed using Bruker TopSpin software, version 3.2. The analysis was performed in duplicate.

##### Protein Content Estimation

The total protein content of the purified biopolymers was assessed using Pierce BCA Protein Assay Kits (Thermo Fisher Scientific, MA, USA) for 10 mg/mL solutions of each sample, according to the manufacturer’s protocol, and the absorbance was measured at 562 nm. The protein concentrations were calculated using a calibration curve prepared with different concentrations (25–2000 μg/mL) of bovine serum albumin.

##### Sugar Analysis by High-Performance Liquid Chromatography (HPLC)

The sugar composition of the purified biopolymers was analyzed according to Gil et al.^29^ About 4 mg of each lyophilized sample was dissolved in 4 mL of deionized water and hydrolyzed with 100 μL of trifluoroacetic acid (Sigma-Aldrich, Missouri, USA) at 120 °C for 5 h. The samples were filtered using 0.2 μm pore size regenerated cellulose syringe filters and transferred to specific vials for HPLC analysis. A CarboPac PA10 column (250 × 4 mm, ThermoFisher Scientific Dionex, California, USA), coupled with an AminoTrap (50 × 4 mm, ThermoFisher Scientific Dionex, California, USA) was used, with a column temperature of 25 °C. The mobile phase used was 18 mM NaOH, with a flow rate of 1 mL/min. Glucose (Scharlau, Barcelona, Spain), d-(+)-galactose, l-rhamnose monohydrate (Fluka Chemie GmbH, Seelze, Germany), d-glucuronic acid, d-galacturonic acid (Alfa Aesar, Massachussets, USA), arabinose, xylose, and mannose (Sigma-Aldrich, Missouri, USA) were used as the standards at concentrations between 0.001 and 0.5 g/L. Duplicate samples were used for the analyses.

##### Fatty Acid Methyl Esters (FAME) Composition and Gas Chromatography–Mass Spectrometry (GC-MS)

To 4 mg of each sample, 2 mL of acidic methanol and 2 mL of ethyl benzoate in chloroform were added, and the tubes were incubated in a silicon bath at 100 °C for 4 h. After 4 h, the tubes were allowed to cool, and 2 mL of hexane was added to each tube and vortexed for 1 min. From the separated phases, 1 mL of the hexane (upper) layer was transferred into a clean glass test tube along with 1 mL of deionized water, followed by vortexing. This washing step was repeated once more. The pH of the hexane phase was checked, ensuring it is neutral, after which 500 μL of the washed hexane phase was transferred to a GC vial for analysis.

An Agilent 6890N GC (Agilent Technologies, Inc., California, USA) equipped with an Agilent 5973 MS detector (with MSD ChemStation, MassHunter Qualitative Analysis 10.0; Agilent Technologies, Inc., California, USA) was used for GC-MS analysis. The GC was equipped with an Agilent J&W HP-5 ms capillary column, 30 m in length with an internal diameter of 0.25 mm (Agilent Technologies, Waldbronn, Germany). The stationary phase of the column was (5%-phenyl) methylpolysiloxane with a film thickness of 0.25 μm. Helium (purity 6.0, 99.9%) was used as the carrier gas. Hexane was used as a rinsing solvent with three pre- and postinjection rinses and two pump cycles. Then, 1 μL of each sample was injected in splitless mode, with a split vent time of 1.2 min. The GC inlet temperature was set at 280 °C. The oven temperature was initially maintained at 80 °C for 1 min and ramped up to 160 °C at 10 °C/min. This was followed by a second ramp up to 270 °C at 5 °C/min, with a hold time of 3 min to ensure separation of long-chain FAMEs. The run time for each sample was 34 min, with the addition of a postrun step at 300 °C for 7 min to recondition the column, bringing the total cycle time to 41 min. The MS was operated in full scan mode with a solvent delay of 3 min, over a mass range of 100–600 *m*/*z*. The detected FAMEs were identified using NIST MS Search 2.2 software (National Institute of Standards and Technology (NIST), Maryland, USA) and by comparison of spectra and retention times with a commercial FAME mix (Supelco 37 Component FAME Mix, Sigma-Aldrich, Taufkirchen, Germany) injected under the same conditions. The total ion chromatograms were visualized using Agilent MassHunter Qualitative Analysis 10.0 software.

##### Thermogravimetric Analyses (TGA)–Differential Thermogravimetric Analyses (DTG)

The thermal degradation behaviors of the purified biopolymers were analyzed using a Thermogravimetric Analyzer Labsys EVO (Setaram, Lyon, France). The lyophilized samples were placed in aluminum crucibles and heated in the presence of air from room temperature to 650 °C, at a heating rate of 10 °C/min.

##### Molecular Weight Determination by Gel Permeation Chromatography (GPC)

The molecular weight of the lyophilized biopolymers was determined using gel permeation chromatography. Prior to analysis, the samples were dissolved in the mobile phase (0.1 M NaNO_3_) and filtered through a 0.22 μm syringe filter. GPC was performed on an HPLC system equipped with a refractive index detector. Data acquisition was done using Clarity chromatography software (DataApex, Prague, Czech Republic) with the GPC Extension module enabled for the analysis of polymers. The column used was a Polysep GFC-P Linear 300 × 7.8 mm (Phenomenex, Torrance, CA, USA). The column temperature was maintained at 40 °C, and 50 μL of each sample was injected. Pullulan standards (Shodex Resonac Europe, Munich, Germany) of known molecular weights were used as standards to calculate the number-average molecular weight (*M*_n_) and weight-average molecular weight of the biosurfactants (*M*_w_). Polydispersity index of the samples was calculated using the following formula:

$$\mathrm{Polydispersity}\;\mathrm{index}=\frac{M_{w}}{M_{n}}$$ 2

### Activity Analysis of Biopolymers

#### CTAB-Methylene Blue Agar Assay

To determine the ionic nature of the biopolymers, CTAB-methylene blue agar assay was performed.^31^ Agar plates were prepared by adding 0.78 g of CTAB (VWR International GmbH, Darmstadt, Germany), 0.002 g of methylene blue (Thermo Fisher Scientific, Massachusetts, USA), and 1.5 g of agar (Thermo Fisher Scientific, Waltham, MA, USA) to 1 L of distilled water (pH 7). Wells (6 mm diameter) were made in the plates, and approximately 150 μL of lyophilized samples from a 1 mg/mL stock prepared in sterile deionized water was added to the wells and incubated at 28 °C overnight. Deionized water was used as a negative control.

#### Hemolysis

To determine whether the biopolymers had hemolytic activity, hemolysis assay was performed.^32^ Approximately 150 μL of lyophilized samples from a 1 mg/mL stock was added to wells in sheep blood agar plates [5% (v/v) defibrinated sheep blood (Sigma-Aldrich, Missouri, USA), blood agar base (VWR International GmbH, Darmstadt, Germany); bacteriological agar 1.2%]. The plates were incubated at 28 °C overnight.

#### Drop-Collapse Assay

Drop-collapse tests using crude oil were performed.^33^ Briefly, 5 μL of crude oil was coated on the well-markings on the lid of a 96-well polystyrene microplate and allowed to equilibrate for 24 h at room temperature (21 ± 1 °C) in a fume hood. Approximately 2 μL of lyophilized biopolymers from a 1 mg/mL stock was transferred to the coated areas, and the drop was observed. Deionized water was used as a negative control, while 1% CTAB was used as the positive control. The results were recorded after 10 min.

#### Emulsification

The ability of the lyophilized biopolymers to emulsify crude oil and engine oil was measured according to Rani et al.^34^ and Cirigliano and Carman,^35^ with minor modifications. Briefly, 500 μL of each biosurfactant solution was diluted with 5 mL of phosphate-buffered saline (Sigma-Aldrich, St. Louis, MμA, USA; pH 7). Then, 5 mg of crude oil or engine oil was added to the mixture, and the mixture was vortexed for 1 min. The emulsion mixture was allowed to settle for 20 min, following an optical density measurement using a UV–visible spectrophotometer at 610 nm (Macherey-Nagel Nanocolor UV/vis II, Duren, Germany). Appropriate blanks of crude oil and engine oil with no biosurfactant were used. One unit of emulsification activity was defined as the amount of biosurfactant that formed an emulsion with an absorbance of 1.0 at 610 nm.

#### Surface Activity

The surface tension-lowering ability of the lyophilized biopolymers was estimated using the pendant drop method in a tensiometer (Krüss Advance, Hamburg, Germany). For calculation of the critical micelle concentration, stocks of 0.1–5 mg/mL concentrations of each sample were prepared, and the surface tension was plotted against concentration. The critical micelle concentration was considered to be the minimum concentration at which maximum surface tension reduction is achieved.

### Statistical Analyses

All of the experiments were conducted in triplicate, unless otherwise mentioned. The results are expressed as the mean ± standard deviation.

## Results and Discussion

Biosurfactant research at the laboratory scale is progressing at a remarkable pace, resulting in the commercialization of rhamnolipids, sophorolipids, and mannosylerythritol lipids, to name a few biosurfactant types. However, continued research into new biosurfactants is essential because of the requirement of structural and functional diversity, which is suitable for very different industrial applications. It could be economically advantageous if such biosurfactants could be produced on inexpensive, waste-derived media. A recent techno-economic study concluded that the substrate-related costs were the most important factors affecting economic assessment results, and therefore, raw materials with lowest costs are desired.^36^Conventionally, biosurfactant production requires both hydrophilic and hydrophobic carbon sources such as glucose and rapeseed oil. The market values of glucose and rapeseed oil are USD 593.3/metric ton and USD 1247.3/metric ton, respectively.^37,38^ In comparison, chemically untreated mixed wood waste and food waste are often available for free, while they require disposal fees (USD 23–117/ton) for landfilling.^39,40^

We have previously reported the feasibility of using torrefied wood waste as a nutrient source for the microbial production of biosurfactants by *B. thailandensis* E264 in a consolidated bioprocessing approach.^19^ In the present study, we employed a covalorization strategy and used hydrolysates of three types of waste—torrefied wood waste, food waste, and a mix of both—as growth media to demonstrate biosurfactant production feasibility and understand the effects of these different feedstocks on the structural and functional properties of the biosurfactants produced. On all three waste-derived media, *B. thailandensis* E264 was able to simultaneously produce two distinct types of biosurfactants—rhamnolipids, and a high-molecular-weight biopolymer, which we characterized thoroughly, as described in the following sections.

### Rhamnolipid Characterization

Rhamnolipids extracted from T225, T225–FWS, and FWS waste-derived media had the respective yields of 0.9 ± 0.0, 0.7 ± 0.02, and 0.7 ± 0.01 g/L. These extracts were analyzed for their structural composition by FTIR. The presence of rhamnolipid congeners were confirmed by LC-QQQ-MS/MS. FTIR analysis of the rhamnolipids extracted from T225, FWS, and T225–FWS media revealed a considerable number of similar functional groups (Figure 1) as different rhamnolipids reported in previous studies.^41–43^ T225–FWS rhamnolipids had a slight peak at 3318.6 cm^–1^, possibly depicting O–H stretching bands originating from inter- and intramolecular hydrogen bonding.^41^ This peak was less evident in T225 and FWS rhamnolipids. For rhamnolipids produced in T225–FWS medium, the asymmetric and symmetric stretchings of C–H groups, such as those in the lipid chains of rhamnolipids, were detected at 2921 and 2852 cm^–1^, respectively. For rhamnolipids produced in FWS medium, the asymmetric stretching peaks were found at 2963 and 2918 cm^–1^_,_ whereas the symmetric stretching peak was found at 2850 cm^–1^.^44^ The rhamnolipid produced in T225 did not exhibit symmetric stretching peaks as prominently; however, a peak at 2963 cm^–1^ represented CH_2_ asymmetric stretching vibrations.^41^ T225–FWS rhamnolipids exhibit a sharp peak at 1730 cm^–1^, which confirms the presence of ester carbonyl (C=O) stretching vibration from lipids and fatty acids; T225 rhamnolipids have this peak at 1745 cm^–1^, depicting the ester linkage in rhamnolipid molecules. In FWS rhamnolipids, on the other hand, this peak is present at a slightly lower wavenumber 1714 cm^–1^.^45,46^ In the fingerprint region, vibrations due to C–H and O–H deformation, which are characteristic of carbohydrates such as rhamnose, are found at 1374 and 1258 cm^–1^ (a sharp peak) in T225; 1456, 1405, and 1260 cm^–1^ (a sharp peak) in T225–FWS; and 1411 and 1258 (a sharp peak) in FWS. Another characteristic of glycosidic and alcohol groups in rhamnoses, the C–O stretching vibrations are found at 1080 and 1012 cm^–1^ in T225 and FWS and at 1199, 1116, 1051, and 1029 cm^–1^ in T225–FWS rhamnolipids. The lower range of the fingerprint region (below 1000 cm^–1^), typically representing various rocking vibrations in the lipid structure, was not allocated more closely to avoid ambiguity.

**Figure 1:**
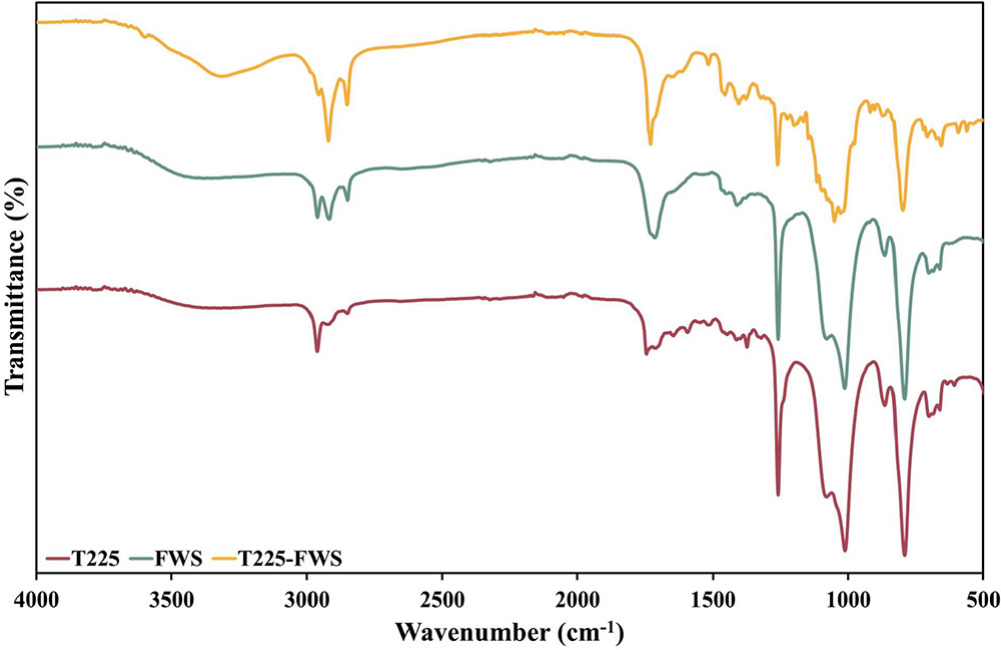
FTIR spectra of rhamnolipids synthesized by *B. thailandensis* E264 from waste-derived media.

The crude rhamnolipid extracts produced in the three different media were analyzed by LC-MS and then by LC-MS/MS to confirm the presence of rhamnolipid congeners; the identification was based on retention times, characteristic adducts, and fragmentation pattern in both positive and negative ionization modes. In all the samples, [M – H]^−^ adducts were observed in the negative mode, whereas [M + Na]^+^ adducts were predominant in the positive mode, with some [M + H]^+^ adducts. The use of positive and negative modes of ionization provided complementary information that improved the congener identification.

The fragmentation of the rhamnolipids has a well-established pattern in both ionization modes. In the case of negative ion mode, peaks of deprotonated rhamnose (*m*/*z* 163), deprotonated dirhamnose (*m*/*z* 309), as well as deprotonated fatty acid chains were present.^47,48^ In case of sodium adducts, the fragmentation included sodiated rhamnose residue (*m*/*z* 169), sodiated rhamnose unit (*m*/*z* 187), and so on.^49^

There were considerable differences in the total ion chromatograms of the extracts. T225–FWS had the highest number of identified rhamnolipid congeners (13), closely followed by FWS (12), whereas T225 contained only three distinct dirhamnolipid congeners (Table 2). These included Rha–Rha–C10–C14/C14–C10, Rha–Rha–C12–C14/C14–C12, and Rha–Rha–C14–C14. In sharp contrast, T225–FWS and FWS contained a mix of mono- and dirhamnolipids, with the absence of the monorhamnolipid Rha–C14–C16/Rha–C16–C14 in FWS being the only difference in the two rhamnolipid crude extracts. T225–FWS and FWS also contained three congeners with double bonds in one of the fatty acid chains (Rha–Rha–C12–C14:1/C14:1–C12, Rha–Rha–C14:1–C14/C14–C14:1, and Rha–Rha–C14–C16:1/C16:1–C14), which were not present in the flash-chromatography-purified extract of rhamnolipids derived from medium containing glycerol, which was used as a standard. It is important to emphasize that isomeric species such as Rha–Rha–C10–C14 and Rha–Rha–C14–C10 cannot be separated chromatographically; therefore, we report them as Rha–Rha–C10–C14/C14–C10 and so on. A representative MS spectrum of Rha–Rha–C14–C14 is illustrated in Figure 2.

**Figure 2:**
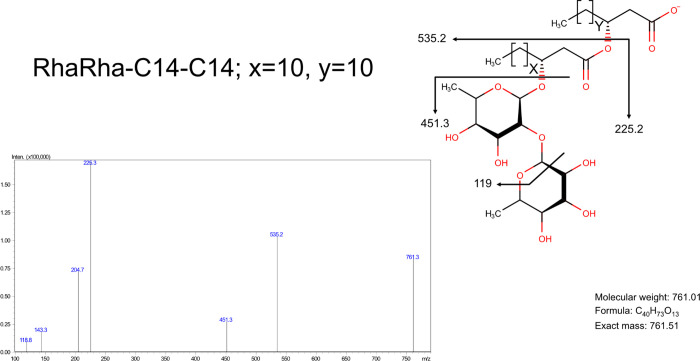
Representative MS spectra of the dirhamnolipid Rha–Rha–C14–C14 produced from waste-derived media by *B. thailandensis* E264.

**Table 2:** Identified Congeners in Rhamnolipid Extracts Synthesized by *B. thailandensis* E264 from Different Waste-Derived Media

no.	compound	retention time	present in standard at same retention time	[M – H]^−^	[M + H]^+^	[M + Na]^+^
T225						
1.	unidentified	0.9	no	417		441
2.	unidentified	6.8	no	282	284	
3.	Unidentified	7.6	no	313	315	
4.	unidentified	8.1	no	307		331
5.	Rha–Rha–C10–C14/C14–C10	14.1	yes	705		729.3
6.	Rha–Rha–C12–C14/C14–C12	16.6	yes	733.3		757.4
7.	Rha–Rha–C14–C14	18.9	yes	761		785
T225–FWS						
1.	unidentified	0.9	no	417		441
2.	unidentified	3.8	yes	716	718	
3.	unidentified	6.5	yes	698		722
4.	unidentified	6.8	no	282	284	
5.	Rha–Rha–C8–C14/C14–C8	11.7	yes	677		701
6.	Rha–C8–C14/C14–C8	13.5	yes	531		555
7.	Rha–Rha–C10–C14/C14–C10	14.1	yes	705		729.3
8.	Rha–Rha–C12–C14:1/C14:1–C12	14.9	no	731		755.4
9.	Rha–C10–C14/C14–C10	15.9	yes	559		583
10.	Rha–Rha–C12–C14/C14–C12	16.6	yes	733.3		757.4
11.	unidentified	17.4	no	805		829
12.	Rha–Rha–C14:1–C14/C14–C14:1	17.4	no	759		783
13.	Rha–C12–C14/C14–C12	18.3	no	587		611.3
14.	unidentified	18.4	no	659		683
15.	Rha–Rha–C14–C14	19	yes	761		785
16.	Rha–Rha–C14–C16:1/C16:1–C14	19.5	no	787.3		811.4
17.	unidentified	19.6	no	833		857
18.	Rha–C14–C14	20.4	yes	615		639
19.	Rha–Rha–C14–C16/C16–C14	21.1	yes	789		813
20.	Rha–C14–C16/C16–C14	22.3	no	643		667
FWS						
1.	unidentified	0.8	no	417		441
2.	unidentified	6.8	no	282	284	
3.	Rha–Rha–C8–C14/C14–C8	11.7	yes	677		701
4.	Rha–C8–C14/C14–C8	13.4	yes	531	533	
5.	Rha–Rha–C10–C14/C14–C10	14.1	yes	705		729.3
6.	Rha–Rha–C12–C14:1/C14:1–C12	15	no	731		755.4
7.	Rha–C10–C14/C14–C10	16	yes	559		583
8.	Rha–Rha–C12–C14/C14–C12	16.6	yes	733.3		757.4
9.	unidentified	17.4	no	805		829
10.	Rha–Rha–C14:–C14/C14–C14:1	17.4	no	759		783
11.	Rha–C12–C14/C14–C12	18.3	no	587		611.3
12.	Rha–Rha–C14–C14	19	yes	761		785
13.	Rha–Rha–C14–C16:1/16:1–14	19.5	no	787.3		811.4
14.	Rha–C14–C14	20.4	yes	615		639
15.	Rha–Rha–C14–C16/C16–C14	21.1	yes	789		813

Media-dependent differences in congener profiles were evident in the rhamnolipid profiles—media containing food waste had a more diverse profile of rhamnolipids than media containing wood hydrolysate alone. This could be due to nutrient complementarity, wherein food waste hydrolysates provide specific precursors or cofactors, resulting in differences in the congener profiles. Wood hydrolysates contain sugars such as glucose, xylose, and mannose; however, they do not contain other nutrients and precursors for metabolic pathways such as amino acids and fatty acids, which are abundant in food waste.^13,43^ When only wood hydrolysates are present in the media, the microorganism synthesizes only a limited number of fatty acids via de novo fatty acid synthesis, which are then incorporated into dirhamnolipids. Typically, monorhamnolipids are synthesized first. Under nutrient-limiting conditions, it is probable that a metabolic shift could have occurred redirecting the resources from growth to secondary metabolite production, such as glycosylation of monorhamnolipids to form dirhamnolipids. *B. thailandensis* tend to produce longer chain fatty acids, with more dirhamnolipids than monorhamnolipids.^28^ Rhamnolipid synthesis in *B. thailandensis* involves three different genes—RhlA, B, and C.^47^ RhlA gene product catalyzes the formation of 3-hydroxyalkanoic acid dimers from two molecules of β-hydroxydecanoyl-acyl carrier protein, which is an intermediate in de novo fatty acid synthesis.^50,51^ RhlB forms a monorhamnolipid by adding the first rhamnose moiety to the 3-hydroxyalkanoyl dimer, and RhlC adds another rhamnose moiety to the monorhamnolipid to form a dirhamnolipid.^52,53^ Further, it has been proven that the composition of mono- and dirhamnolipids can be changed by altering the expression levels of the genes RhlB and RhlC involved in glycosylation of the fatty acid chains during rhamnolipid synthesis.^13,54^ When food hydrolysates or a combination of wood and food hydrolysates is present in the microbial growth media, the nutrient profile appears to be more diverse, resulting in a rhamnolipid profile containing both mono- and dirhamnolipid congeners, including unsaturated fatty acid chains.^43^ There are previous reports of mixed waste hydrolysates resulting in different proportions of rhamnolipid congeners; however, there is not much mechanistic explanation for the phenomenon. For instance, more hydrophobic rhamnolipid congeners were observed in waste-derived media containing sugar cane molasses, corn steep liquor, and oil mill wastewater compared to media without oil mill wastewater.^55,42^ Other plausible explanations in literature may be that the combination of wood and food waste hydrolysates could result in specific C:N ratios, triggering a moderate level of metabolic stress resulting in biosurfactant production associated with quorum sensing.^56^ However, these assumptions can be conclusively proved only using transcriptomic analyses or isotope-labeled carbon and nitrogen source tracing.

In addition to the identified rhamnolipid compounds, some other compounds that could not be identified were also detected, which are also listed in Table 2.

### Physicochemical and Functional Properties of Biopolymers

Besides the rhamnolipids that were extracted via liquid–liquid extraction, *B. thailandensis* also produced certain high-molecular-weight waste biopolymers on all three waste-derived media, which could be separated and concentrated via ultrafiltration. The biopolymers produced by *B. thailandensis* on T225, T225–FWS, and FWS media were quantified on the basis of weight of lyophilized samples as 0.3 ± 0.01, 0.9 ± 0.02, and 1.1 ± 0.02 g/L, respectively. These values represent the crude biopolymer fractions rather than purified glycolipopeptides. These were comprehensively characterized and tabulated (Table 3).

**Table 3:** Physicochemical and Functional Properties of Biopolymers Synthesized by *B. thailandensis* E264 from Different Waste-Derived Media Composition

high molecular weight biopolymer	T225	T225–FWS	FWS
yield (g/L)	0.3 ± 0.01	0.9 ± 0.02	1.1 ± 0.02
C (%)	46.3 ± 0.2	49.1 ± 0.1	43.9 ± 0.1
H (%)	7.1 ± 0.0	7.6 ± 0.0	6.5 ± 0.2
N (%)	7.1 ± 0.0	5.8 ± 0.0	6.4 ± 0.0
S (%)	0.5 ± 0.0	0.3 ± 0.0	0.4 ± 0.0
O (%)	39.0 ± 0.1	37.2 ± 0.1	42.8 ± 0.3
protein (mg/g of biopolymer)	94.1 ± 0.0	82.5 ± 0.0	92.4 ± 0.0
number-average molecular weight (*M*_n_) (Da)	5.1 × 10^5^	3.3 × 10^5^	3.7 × 10^5^
weight-average molecular weight (*M*_w_) (Da)	5.5 × 10^5^	4.4 × 10^5^	5.0 × 10^5^
polydispersity index	1.1	1.3	1.3
emulsification activity with crude oil (OD_610 nm_)	2.0	2.1	1.2
emulsification activity with engine oil (OD_610 nm_)	2.4	1.6	1.9
critical micelle concentration (mg/mL)	1.2	1.1	0.7
surface tension at critical micelle concentration (mN/m)	36.8	35.4	48.5

### Compositional Studies

Elemental composition of all three biosurfactants revealed the presence of carbon, hydrogen, nitrogen, and sulfur (Table 3). The presence of nitrogen could be indicative of amide bonds in peptides.^57,58^ Traces of sulfur suggest the presence of amino acids such as cysteine, which could be incorporated into peptides or proteins in these biopolymers.^59^

The FTIR patterns of the three lyophilized biopolymers were relatively similar to one another, with minor differences (Figure 3). For all three biopolymers, a broad peak (3284, 3286, and 3282 cm^–1^, respectively, for T225, T225–FWS, and FWS biopolymers) was present, which could represent the O–H stretch of sugars (typically found between 3600 and 3000 cm^–1^) or the N–H stretch of peptide bonds (typically at 3300–3100 cm^–1^). There is a considerable overlap between these two types of bonds, primarily because of the presence of extensive hydrogen bonding in both the bonds.^60^ The presence of lipid groups could be confirmed by asymmetric stretching vibrations of C–H groups at 2921, 2924, and 2925 cm^–1^ for T225, T225–FWS, and FWS biopolymers, respectively. Additionally, symmetric stretching vibrations of aliphatic chains were found in T225 and T225–FWS at 2852 and 2854 cm^–1^, respectively.^44^ For the FWS, the symmetric stretching vibration in this region was much less evident. Glycation of proteins or the presence of sugars could also be deduced by the clear presence of sharp peaks at 1040 cm^–1^ for T225 and T225–FWS and at 1036 cm^–1^ for FWS.^61^ The presence of the peptide moiety was confirmed by the amide I band representing C=O stretching in the backbone at 1640, 1641, and 1635 cm^–1^, respectively, for T225, T225–FWS, and FWS biopolymers.^62,63^ Amide I peak ranges of 1640–1612 cm^–1^, such as those mentioned above, could even denote the presence of protein secondary structures such as β-sheets with high accuracy.^64^ Further, amide II bonds representing N–H bending and C–N stretching vibrations were present at 1539 cm^–1^ for T225 and FWS and at 1538 cm^–1^ for FWS biosurfactants, in addition to less intense amide III bonds (also representing C–N stretching and N–H bending vibrations) at 1241, 1238, and 1239 cm^–1^, respectively, for T225, T225–FWS, and FWS biopolymers.^62,63^

**Figure 3:**
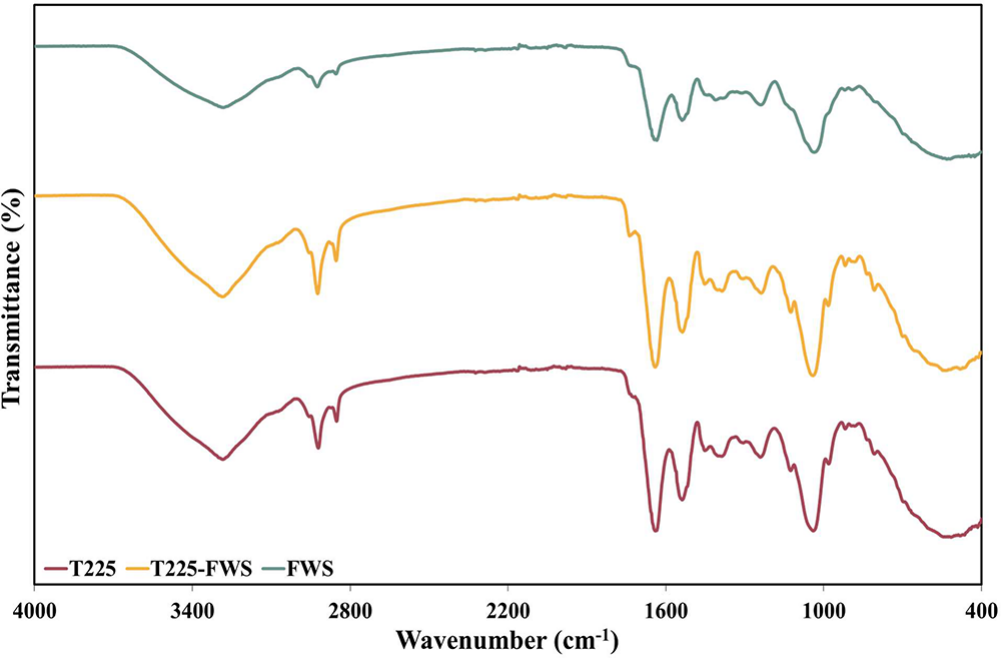
FTIR spectra of biopolymers synthesized by *B. thailandensis* E264 from waste-derived media.

The spatial arrangement of protons in the biopolymers was studied using ^1^H NMR (Figure 4) and interpreted in comparison with relevant studies. T225, T225–FWS, and FWS biopolymers exhibited moderately similar spectra with differences in intensity. Prominent peaks indicating chemical shifts at 0.8–1.2 ppm were attributed to terminal methyl groups (−CH_3_) and methylene chains (−(CH_2_)*n*), suggesting the presence of long-chain fatty acids.^65–67^ Similar strong signals that may be methylene protons (−(CH2)*n*) were observed at 1.9–2.5 ppm for T225–FWS and FWS and at 2.3–2.5 for T225.^68^ Moderate signals which could correspond to protons within the sugar ring were observed at 3.6–3.9, 3.5–4.2, and 3.5–3.9 ppm, respectively, for T225, T225–FWS, and FWS.^68^ Moderate signals in the region 4.5–5.2, 4.7–5.3, and 4.5–5.3 ppm for T225, T225–FWS, and FWS, respectively, are likely to be anomeric protons in the sugar moieties, as well as olefinic protons suggesting minor unsaturation (typically 5.3–5.5).^68^ The midfield region (particularly 4–5 ppm) could also represent α protons from amino acids, according to established previous reports, presenting an overlap region for different types of protons.^68–70^ All three biopolymers also exhibited minor to moderately intense signals in the 6.6–8.4 ppm region that could be attributed to amide protons or aromatic amino acid protons.^68,66,65^ These observations and literature comparisons suggest that all biopolymers are glycolipopeptides.

**Figure 4:**
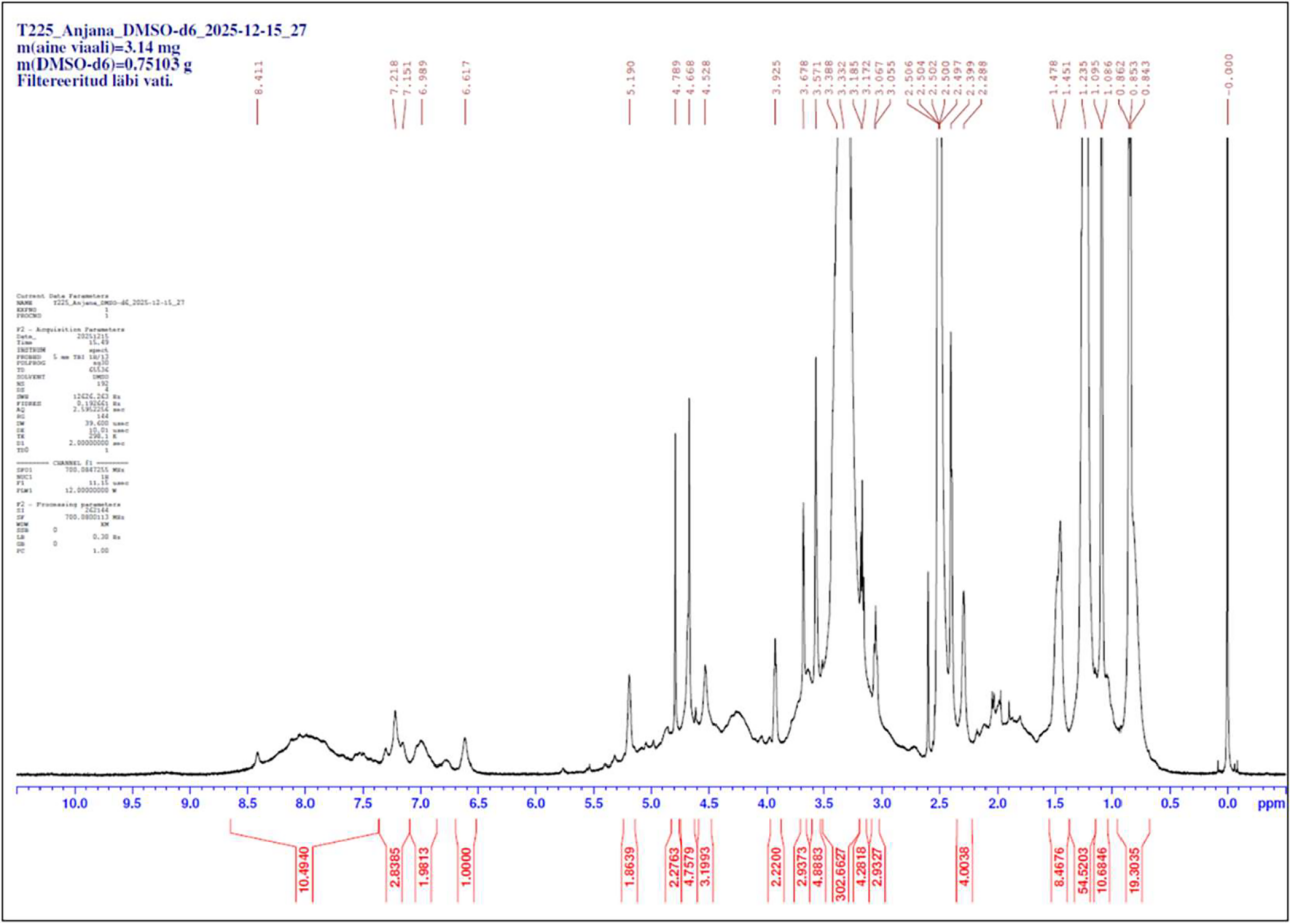
Representative ^1^H NMR Spectra of the T225 biopolymer.

Based on the analyses mentioned above, it was assumed that the biopolymers also contained proteins or peptides in their structure, and the protein contents of T225, T225–FWS, and FWS biosurfactants were estimated to be low at 94.1 ± 0.0, 82.5 ± 0.0, and 92.4 ± 0.0 mg/g of biopolymer, respectively, corroborating the NMR results.

The carbohydrate components of the biopolymers were identified by HPLC after acid hydrolysis. T225–FWS and FWS were composed of rhamnose, arabinose, galactose, glucose, and xylose, while arabinose was absent in T225, suggesting that the arabinose component was derived from food waste hydrolysate rather than torrefied wood hydrolysate. Indeed, arabinose, being a hemicellulosic side-chain sugar, is among the first components to be degraded during torrefaction.^71^ There were notable differences in the relative proportions of these sugars in each biosurfactant. Whereas galactose was the most abundant sugar moiety in FWS biopolymer, rhamnose was present in the highest concentration in T225 and T225–FWS. The percentage compositions are presented in Figure 5, depicting medium-dependent compositional changes. Further, the relative proportions of hexoses to pentoses were similar in biopolymers derived from media containing wood hydrolysates (77:23 and 76:24 in T225 and T225–FWS, respectively), whereas they were 61:39 in FWS biopolymers.

**Figure 5:**
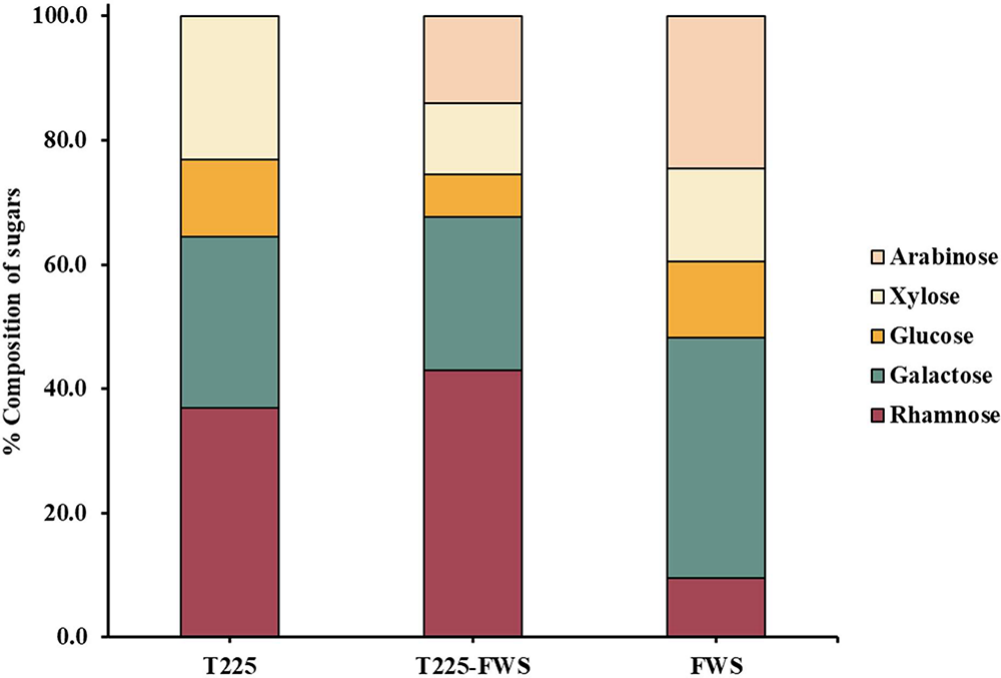
Sugar composition of biopolymers synthesized by *B. thailandensis* E264 from waste-derived media.

The lipid components of the biopolymers were identified based on the retention time and retention index of methyl-esterified fatty acids compared with a FAME mix standard. A mix of saturated and unsaturated fatty acids with carbon chain length ranging from 12 to 18 were detected in all three biosurfactant extracts, with a relatively similar profile (Table 4). However, FWS biosurfactant also contained pentadecanoic acid and heptadecanoic acid, which are both odd-carbon-chain fatty acids. As mentioned in the previous section, the biosynthetic pathway of rhamnolipids in *B. thailandensis* has been well characterized. Only one other glycolipopeptide has been reported previously in this organism, and hence, there is no information about the biosynthetic pathway of the newly identified glycolipopeptide biosurfactants in this study.^29^ However, the de novo fatty acid synthesis pathway is probably the main contributor of lipid chains based on the similar profile of lipid composition across all three biopolymers.

**Table 4:** Identified Fatty Acid Methyl Esters in Biopolymers T225, T225–FWS, and FWS

no.	compound	retention time (min)	no. of carbon atoms in fatty acid	saturation
T225				
1.	dodecanoic acid, methyl ester	10.8	12	saturated
2.	tetradecanoic acid, methyl ester	13.8	14	saturated
3.	9-hexadecenoic acid, methyl ester	16.9	16	unsaturated
4.	hexadecanoic acid, methyl ester	17.2	16	saturated
5.	9,12-octadecadienoic acid, methyl ester	20.1	18	unsaturated
6.	11-octadecenoic acid, methyl ester	20.3	18	unsaturated
7.	octadecanoic acid, methyl ester	20.7	18	saturated
T225–FWS				
1.	dodecanoic acid, methyl ester	10.8	12	saturated
2.	9-tetradecenoic acid, methyl ester	13.7	14	unsaturated
3.	tetradecanoic acid, methyl ester	13.8	14	saturated
4.	9-hexadecenoic acid, methyl ester	16.9	16	unsaturated
5.	hexadecanoic acid, methyl ester	17.2	16	saturated
6.	9,12-octadecadienoic acid, methyl ester	20.1	18	unsaturated
7.	11-octadecenoic acid, methyl ester	20.3	18	unsaturated
8.	octadecanoic acid, methyl ester	20.7	18	saturated
FWS				
1.	dodecanoic acid, methyl ester	10.8	12	saturated
2.	tetradecanoic acid, methyl ester	13.8	14	saturated
3.	pentadecanoic acid, methyl ester	15.5	15	saturated
4.	9-hexadecenoic acid, methyl ester	16.9	16	unsaturated
5.	hexadecanoic acid, methyl ester	17.2	16	saturated
6.	heptadecanoic acid, methyl ester	18.9	17	saturated
7.	9,12-octadecadienoic acid, methyl ester	20.1	18	unsaturated
8.	11-octadecenoic acid, methyl ester	20.3	18	unsaturated
9.	octadecanoic acid, methyl ester	20.7	18	saturated

### Thermal Degradation Profile of Biopolymers

In TGA, all three biopolymers exhibited a multistep degradation pattern (Figure 6). An initial weight loss of ∼8 to 11% occurred below 150 °C, which is attributed to the evaporation of residual moisture. This observation is consistent with earlier reports, indicating a minor mass loss of biosurfactants (<10%) up to 180 °C, owing to dehydration.^72^ Following this drying phase, the biosurfactants remained stable until the onset of primary decomposition, which occurred between ∼180 and 350 °C, as shown by the sharp weight loss in this range (Figure 6a). The DTG curves also show a prominent peak at approximately 240–300 °C (Figure 6b), indicating that the predominant organic matter degraded within this range. For example, FWS and T225 showed peak DTG decomposition rates at 259 and 281 °C, respectively, with a mass loss of 40–50%. The significant weight loss observed between ∼200 and 340 °C is characteristic of complex biosurfactants and is generally attributed to the degradation of organic components, particularly proteinaceous and polysaccharide constituents bound within surfactants.^73^ In agreement with this observation, the original study on a glycolipopeptide biosurfactant from *B. thailandensis* reported that a major mass loss (∼40%) occurred between 180 and 340 °C, corresponding to the breakdown of proteins and polysaccharide side chains.^29^ Similarly, another study reported that a biosurfactant derived from agro-food waste displayed primary thermal degradation (140 and 347 °C) due to the breakdown of organic functional groups. However, T225–FWS exhibited a two-stage degradation profile, with a secondary DTG peak at ∼526 °C and a primary mass loss at ∼240 to 250 °C. This indicates that the T225–FWS biosurfactant contains thermally stable fractions, such as aromatic/hydrocarbon moieties resistant to decomposition, which are likely derived from the compositional differences in mixed-feedstock fermentation.^74^ Furthermore, the thermostability of T225–FWS was also reflected in retaining a higher residual mass fraction at a higher temperature of approximately 30% at 400 °C, compared to ∼21% for T225 and ∼27% FWS, respectively. However, at 600 °C, even the stable fractions decomposed, leaving all the samples with <7% of their initial mass, corresponding to the inorganic content of the biopolymers and indicating that the sample was purified. These findings indicate that the produced biosurfactants showed thermal stability comparable to that of previously reported rhamnolipid-type glycolipids and lipopeptides (stable up to ∼180 °C), remaining intact up to ∼200 °C, suggesting potential high-temperature applications, after stringent, real-world performance testing.^72–74^

**Figure 6:**
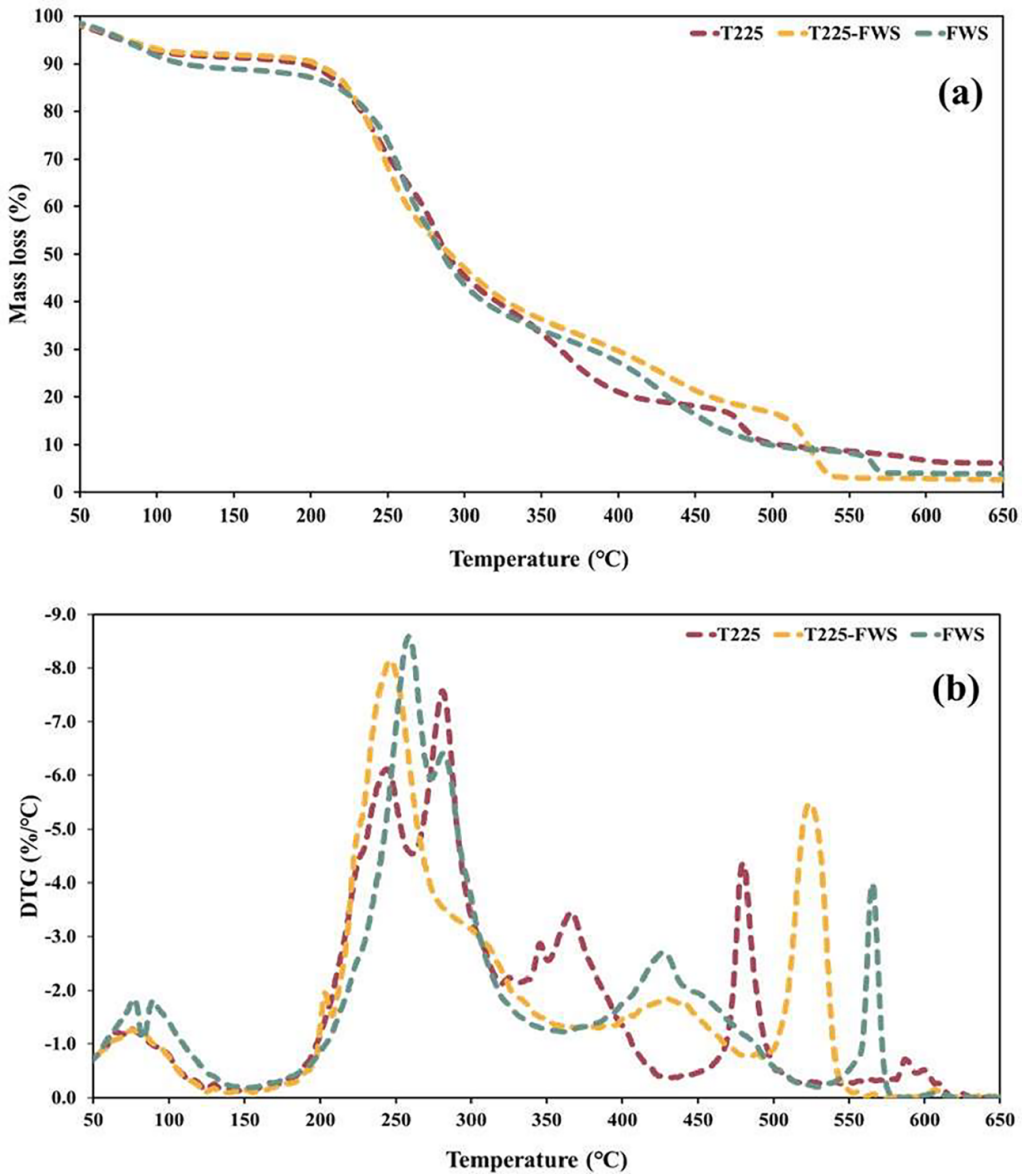
(a) Thermogravimetric analyses and (b) Derivative thermogravimetric analyses (DTG) of biopolymers T225, T225–FWS, and FWS showing multistage thermal degradation.

### Molecular Weight Distribution of Biopolymers

GPC was employed to evaluate the molecular weight distribution of the biosurfactants produced from the T225, T225–FWS, and FWS samples. As summarized in Table 3, the weight-average molecular weights (*M*_w_) and number-average molecular weights (*M*_n_) of the biosurfactants ranged from 4.4 × 10^5^ to 5.5 × 10^5^ Da and 3.3 × 10^5^ to 5.1 × 10^5^ Da, respectively, with polydispersity indices (PDI) between 1.1 and 1.3. These results indicate that all three samples were high-MW biosurfactants with relatively narrow molecular weight distributions.^75^ Among the three, the biosurfactant derived from T225 exhibited the highest *M*_n_ and *M*_w_ of 5.1 × 10^5^ and 5.5 × 10^5^ Da, respectively, with a narrow PDI of 1.1. This low PDI indicates a relatively uniform molecular size distribution. However, the biosurfactants derived from T225–FWS and FWS showed slightly lower *M*_n_ (3.3 and 3.7 × 10^5^ Da) and *M*_w_ (4.4 and 5.0 × 10^5^ Da), respectively. Both biosurfactants displayed a broader PDI of 1.3, suggesting a more heterogeneous mixture of molecular sizes. These differences in molecular weight result from the impact of feedstock composition, which alters microbial metabolism and produces biosurfactants with varying lengths or structures.^76,77^

### Surfactant Activity of High-Molecular-Weight Polymeric Biosurfactants

CTAB is a cationic surfactant that in the presence of methylene blue forms insoluble blue precipitates with anionic species.^31^ The ionic charges of the biopolymers were determined via preliminary screening using the CTAB-methylene blue agar assay. All of the biopolymers produced blue haloes around the wells, indicating a net negative charge (Figure 7a). Hemolytic potential assay is an indicator of membrane disruption potential related to biocompatibility in terms of potential antimicrobial activity. The results of the hemolytic assay revealed that all three newly isolated biosurfactants demonstrated clear β-hemolytic activity, with the size of the lysis zone following a similar trend as in the CTAB-methylene blue agar assay (T225 ≈ T225–FWS > FWS; Figure 7b). A previous study has correlated the length of the acyl chain with the degree of hemolysis, with longer chains inducing a higher degree of hemolysis.^78^ The results of the GC-MS analysis reveal that the biosurfactants contain long-chain fatty acids ranging from C12 to C18, thus validating the degree of hemolysis. However, multiple other factors such as headgroup, CMC, and experimental conditions such as concentration of surfactant, pH, and so on also influence the degree of hemolysis, and our results are reported as qualitative indicators of surfactant activity.

**Figure 7:**
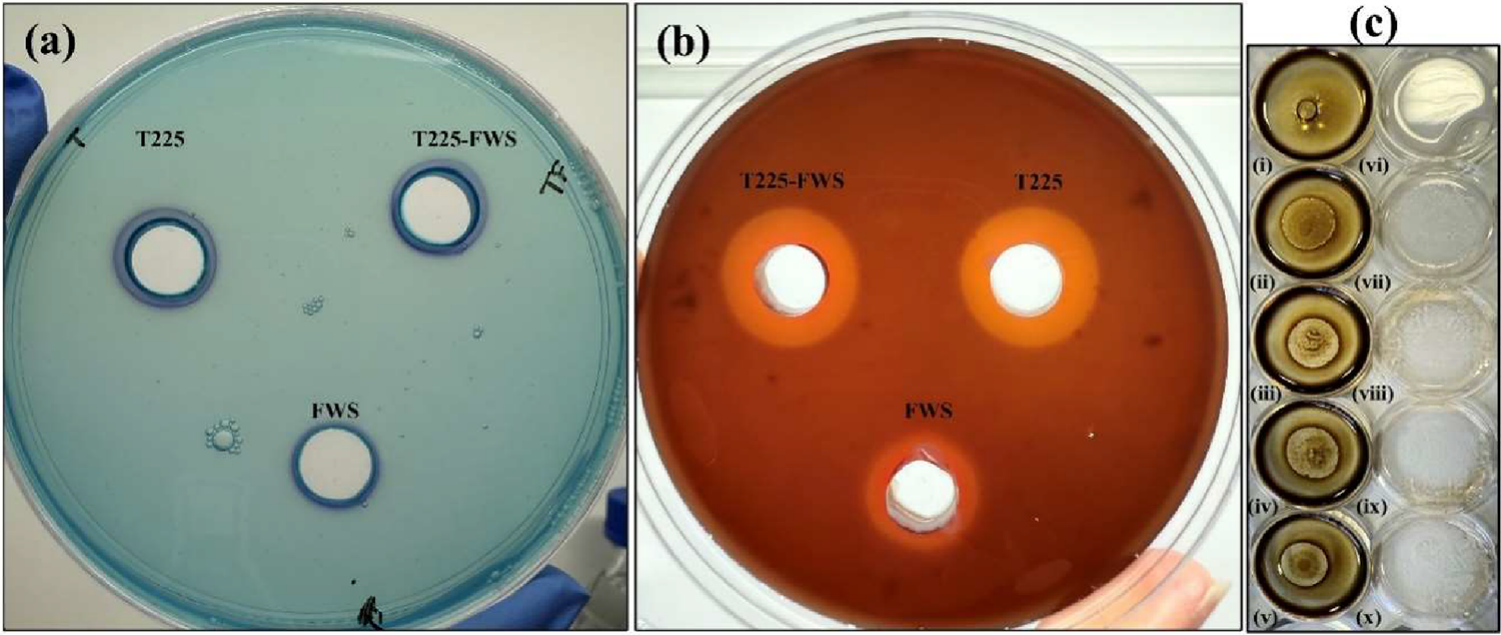
Activity assays of biopolymers: (a) CTAB-methylene blue agar assay of biopolymers; (b) Hemolysis assay of biopolymers; and (c) drop-collapse assay against crude oil (i–v) and engine oil (vi–x) of biopolymers: i, vi, deionized water; ii, vii, 1% CTAB; iii, viii, T225; iv, ix, T225–FWS; v, x, FWS.

In the drop-collapse assay, all three biosurfactants exhibited tensio-activity (Figure 7c). On the crude-oil- coated plate, T225 and T225–FWS biosurfactant drops started spreading almost immediately and collapsed within 7–8 min, indicative of strong interfacial tension-reducing properties.^79^ FWS exhibited slightly delayed activity, taking approximately 10 min to completely collapse. In comparison, the positive control, CTAB, collapsed within 4–5 min, whereas the water drop remained intact for the duration of the experiment due to the absence of surface-active compounds.

OD at 610 nm is typically used as a measure of the turbidity of emulsions, as it avoids interference from colored components and indicates the quality of the emulsion.^80^ The biopolymers were able to emulsify crude oil and engine oil well (Table 3), with all three biosurfactants exhibiting OD values greater than 1, suggesting that these biosurfactants were able to disperse the oil into a large number of well-dispersed droplets.^81^ Crude oil emulsification ability was as follows: T225–FWS > T225 > FWS. All three biosurfactants outperformed the positive control, rhamnolipids, in crude oil emulsification, suggesting that these biosurfactants were able to form stable and uniform distribution of oil droplets, with smaller droplet sizes than rhamnolipid.^82,83^ Regarding engine oil, the order of emulsification was T225 > FWS > T225–FWS, with T225–FWS performing slightly lower than the positive control. These results suggest that the biopolymers could find application in oil bioremediation.

For all of the biopolymers, the CMC was calculated on the basis of their ability to reduce the surface tension of water. The tested concentration range ensured sufficient coverage of both pre- and postmicellar regions, resulting in reliable identification of the micellar transition stage; however, higher resolution measurements in the CMC region would lead to enhanced precision. As expected, the surface tension-lowering ability increased with an increase in concentration from 0.1 to 5 mg/mL and remained relatively unaltered for the higher concentrations (Figure 8). The CMC values of T225, T225–FWS, and FWS biosurfactants were 1.2, 1.1, and 0.7 mg/mL, respectively (Table 3), which are relatively high, but within the limits reported for similar biosurfactants.^29,43^ Commercial surfactants exhibit a varied CMC range, such as linear alkylbenzenesulfonates (CMC—0.1–0.65) and sodium dodecyl sulfate (CMC—2.3–2.5 mg/mL), whereas in commercial biosurfactants such as rhamnolipid, the CMC often varies in the range of 5–380 mg/mL.^57,84–86^ At their respective CMC levels, T225–FWS had the highest tensio-activity and was able to reduce surface tension to 35.4 mN/m, followed by T225 (36.8 mN/m). The FWS biosurfactant reduced the surface tension to 48.5 mN/m, which is rather high compared to the other two surfactants; however, it is similar to the tensio-activity reported for other glycolipopeptides (values ranging from 40.4 to 69.1 mN/m).^29,77^

**Figure 8:**
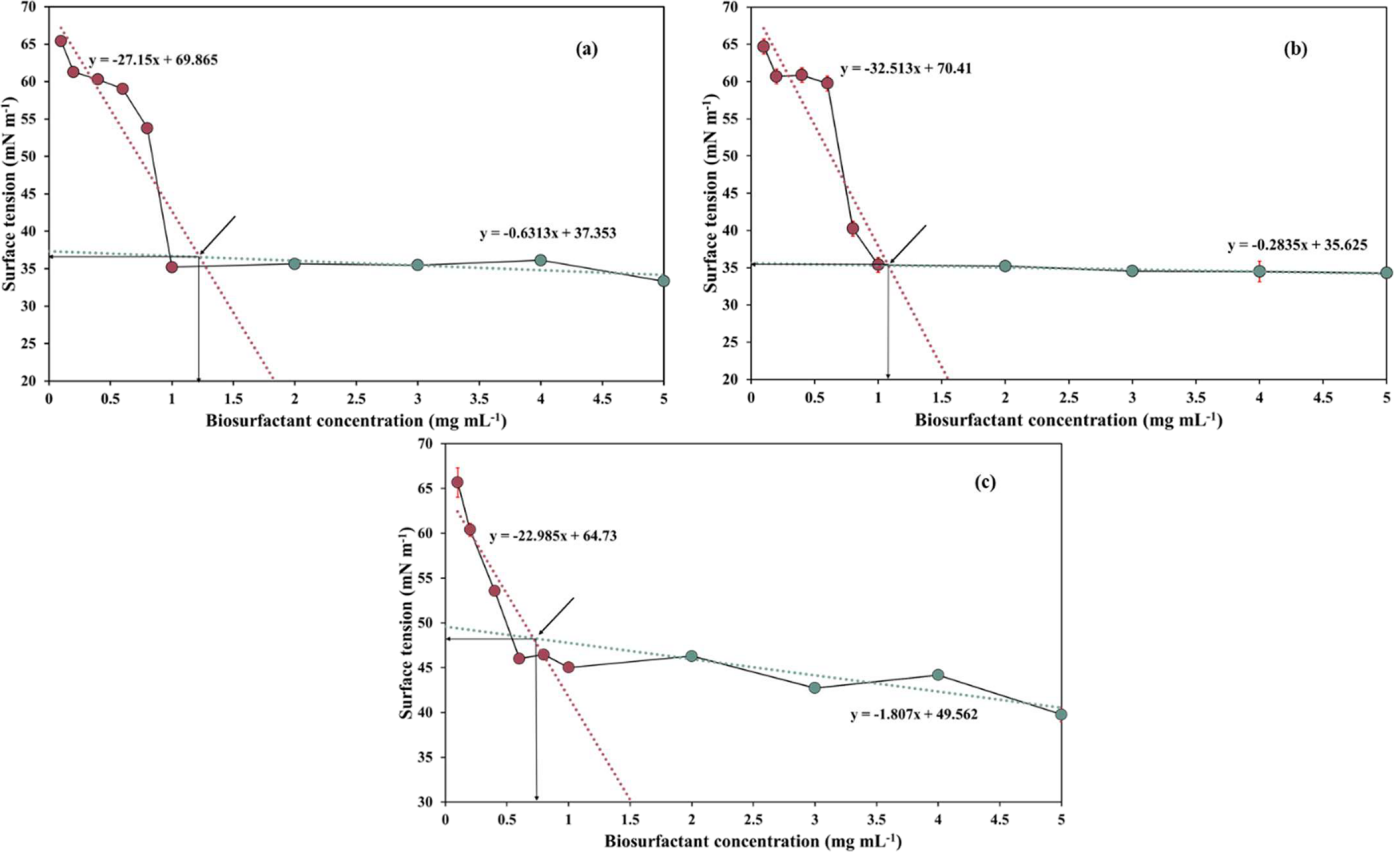
Critical micelle concentration (CMC) of high-molecular-weight biosurfactants from waste-derived media: (a) T225; (b) T225–FWS; and (c) FWS.

Taken together, the aforementioned results suggest that these high-molecular-weight, polymeric biosurfactants derived from three different media formulated from two abundantly available waste-streams have very good surface and interfacial tension-reducing properties as well as β-hemolytic activity, all of which are indicative of surfactant activity. Based on emulsification and drop-collapse assays, the surface and interfacial tension-reducing properties could be of value in oil bioremediation, particularly in areas contaminated with crude oil or engine oil. Moreover, media-dependent changes in the composition reinforce the possibility of designer or tailor-made biosurfactants suitable for various applications following detailed three-dimensional structural and application studies.

## Conclusions and Future Perspectives

While the production of rhamnolipids by *B. thailandensis* E264 has been reported previously in many defined and undefined media formulations, to the best of the authors’ understanding, this is the first preliminary feasibility study describing the simultaneous production of high-molecular-weight polymeric biosurfactants with rhamnolipids in waste-derived media, suggesting potential application in a biorefinery-type operation, pending process optimization and techno-economic process estimations. Torrefaction appears to be an ideal pretreatment solution, as it offers possibilities for the use of mild temperatures, process heat generation via side-stream utilization, and the integration of the existing torrefaction infrastructure to reduce both process costs and initial capital investments. At any rate, considering the high market price of biosurfactants compared to biocoal, detailed exploration of torrefied biomass as a biorefinery feedstock is worth considering.

Both the rhamnolipids and the biopolymers produced from the three waste-derived media showed differences in composition. The crude biopolymer fractions were found to contain glycolipopeptides, with traces of sulfur content present, which are rarely found in nature. Their molecular weights were in the range of 4.4 × 10^5^–5.5 × 10^5^ Da. Minor-to-moderate differences in the sugar and fatty acid composition suggest that although the de novo fatty acid synthesis pathway is probably the main contributor of lipid chains, there is a possibility of generating tailor-made biosurfactant structures suited for specific applications such as bioremediation and emulsification by altering the sugar and fatty acid composition of the production medium, without the need for expensive genetic engineering techniques. All of the polymeric biosurfactants displayed considerable thermostability with T225–FWS being the most thermostable. This biosurfactant could be used in fields with high operational temperatures, such as enhanced oil recovery, oil spill bioremediation, industrial cleaning, and pharmaceuticals and cosmetics requiring sterilization, to name a few, following real-world application studies. While the yields of these new biosurfactants were definitely on the lower side of other similar reported molecules, there is definite potential for improvement of yields using process optimization strategies. Further process optimization is also required in the case of developing polymeric biosurfactant product formulations for high-purity applications. This study adds to our knowledge on biosurfactant production and substrate metabolism of *B. thailandensis* E264 on different low-cost, nutrient-rich, and waste-derived substrates while also providing a potential biorefinery strategy for producing different high-value biosurfactants from abundant wastes.

## References

[1] Gabrielli P., Rosa L., Gazzani M., Meys R., Bardow A., Mazzotti M., Sansavini G. (2023). Net-Zero Emissions Chemical Industry
in a World of Limited Resources. One Earth.

[2] Huo J., Wang Z., Oberschelp C., Guillén-Gosálbez G., Hellweg S. (2023). Net-Zero Transition of the Global Chemical Industry
with CO 2 -Feedstock by 2050: Feasible yet Challenging. Green Chem..

[3] Tijjani
Usman I. M., Ho Y.-C., Baloo L., Lam M.-K., Sujarwo W. (2022). A Comprehensive Review on the Advances of Bioproducts
from Biomass towards Meeting Net Zero Carbon Emissions (NZCE). Bioresour. Technol..

[4] Kugaji M., Ray S. K., Parvatikar P., Raghu A. V. (2025). Biosurfactants:
A Review of Different Strategies for Economical Production, Their
Applications and Recent Advancements. Adv. Colloid
Interface Sci..

[5] Nagtode V. S., Cardoza C., Yasin H. K. A., Mali S. N., Tambe S. M., Roy P., Singh K., Goel A., Amin P. D., Thorat B. R., Cruz J. N., Pratap A. P. (2023). Green Surfactants
(Biosurfactants):
A Petroleum-Free Substitute for Sustainability—Comparison,
Applications, Market, and Future Prospects. ACS Omega.

[6] Kocal J. A., Vora B. V., Imai T. (2001). Production of Linear
Alkylbenzenes. Appl. Catal. A Gen.

[7] Naughton P. J., Marchant R., Naughton V., Banat I. M. (2019). Microbial Biosurfactants:
Current Trends and Applications in Agricultural and Biomedical Industries. J. Appl. Microbiol..

[8] Fernandes, N. A. T. ; Simões, L. A. ; Dias, D. R. Comparison of Biodegradability, and Toxicity Effect of Biosurfactants with Synthetic Surfactants. In Advancements in Biosurfactants Research; Aslam, R. ; Mobin, M. ; Aslam, J. ; Zehra, S. , Eds.; Springer International Publishing: Cham, 2023; pp 117–136.

[9] Guzmán E., Ortega F., Rubio R. G. (2024). Exploring the World of Rhamnolipids:
A Critical Review of Their Production, Interfacial Properties, and
Potential Application.. Curr. Opin. Colloid
Interface Sci..

[10] Marchant, R. ; Banat, I. M. Achieving Commercial Applications for Microbial Biosurfactants. In Biosurfactants for the Biobased Economy; Hausmann, R. ; Henkel, M. , Eds.; Springer International Publishing: Cham, 2022; pp 181–193. 10.1007/10_2021_191 35246696

[11] Liu G., Zhong H., Yang X., Liu Y., Shao B., Liu Z. (2018). Advances in Applications of Rhamnolipids
Biosurfactant in Environmental
Remediation: A Review. Biotechnol. Bioeng..

[12] Chauhan S., Mohanty A., Meena S. S. (2025). Unlocking
the Potential of Rhamnolipids:
Production via Agro-Industrial Waste Valorization, Market Insights,
Recent Advances, and Applications. Biomass Conv.
Bioref..

[13] Chong H., Li Q. (2017). Microbial Production
of Rhamnolipids: Opportunities, Challenges and
Strategies. Microb. Cell Fact..

[14] Mohanty S. S., Koul Y., Varjani S., Pandey A., Ngo H. H., Chang J.-S., Wong J. W. C., Bui X.-T. (2021). A Critical Review
on Various Feedstocks as Sustainable Substrates for Biosurfactants
Production: A Way towards Cleaner Production. Microb. Cell Fact.

[15] The Baltic Times. Estonia facilitates the use of wood waste as heating material. https://www.baltictimes.com/estonia_facilitates_the_use_of_wood_waste_as_heating_material/ (accessed 2025-08-04).

[16] European Environment Agency. Country profiles on waste prevention - 2025. https://www.eea.europa.eu/en/topics/in-depth/waste-and-recycling/country-profiles-on-waste-prevention-2025 (accessed 2025–08–04).

[17] de
Oliveira Schmidt V. K., de Souza Carvalho J., de Oliveira D., de Andrade C. J. (2021). Biosurfactant Inducers for Enhanced Production of Surfactin
and Rhamnolipids: An Overview. World J. Microbiol.
Biotechnol..

[18] Khondee N., Ruamyat N., Luepromchai E., Sikhao K., Hawangchu Y. (2022). Bioconversion
of Lignocellulosic Wastes to Zwitterionic Biosurfactants by an Alkaliphilic
Bacterium: Process Development and Product Characterization. Biomass Bioenergy.

[19] Hari A., Rooni V., Veerabagu U., Sarker S., Konist A., Kikas T. (2025). Repurposing Torrefied
Biomass as a Novel Feedstock for Microbial
Bioprocessing—A Proof-of-Concept of Low-Cost Biosurfactant
Production. Polymers.

[20] Ke L., Zhou N., Wu Q., Zeng Y., Tian X., Zhang J., Fan L., Ruan R., Wang Y. (2024). Microwave
Catalytic Pyrolysis of Biomass: A Review Focusing on Absorbents and
Catalysts. npj Mater. Sustain..

[21] Cahyanti M. N., Doddapaneni T. R. K. C., Madissoo M., Pärn L., Virro I., Kikas T. (2021). Torrefaction
of Agricultural and
Wood Waste: Comparative Analysis of Selected Fuel Characteristics. Energies.

[22] Yun H., Wang Z., Wang R., Bi X., Chen W.-H. (2021). Identification
of Suitable Biomass Torrefaction Operation Envelops for Auto-Thermal
Operation. Front. Energy Res..

[23] Haseli Y. (2018). Process Modeling
of a Biomass Torrefaction Plant. Energy Fuels.

[24] What is Torrefaction. https://www.blackwood-technology.com/technology/what-is-torrefaction/ (accessed 2025-11-26).

[25] Chen C., Su J., Ali A., Zhai Z. (2022). Cornstalk Biochar Promoted the Denitrification
Performance and Cellulose Degradation Rate of Burkholderia Sp. CF6. J. Environ. Chem. Eng..

[26] Morya R., Salvachúa D., Thakur I. S. (2020). Burkholderia: An Untapped but Promising
Bacterial Genus for the Conversion of Aromatic Compounds.. Trends Biotechnol..

[27] Morya R., Kumar M., Singh S. S., Thakur I. S. (2019). Genomic Analysis
of Burkholderia Sp. ISTR5 for Biofunneling of Lignin-Derived Compounds. Biotechnol. Biofuels.

[28] Funston S. J., Tsaousi K., Rudden M., Smyth T. J., Stevenson P. S., Marchant R., Banat I. M. (2016). Characterising Rhamnolipid
Production
in Burkholderia thailandensis E264, a Non-Pathogenic Producer. Appl. Microbiol. Biotechnol..

[29] Gil C. V., Rebocho A. T., Esmail A., Sevrin C., Grandfils C., Torres C. A. V., Reis M. A. M., Freitas F. (2022). Characterization of
the Thermostable Biosurfactant Produced by Burkholderia thailandensis
DSM 13276. Polymers.

[30] Marvin - Chemical Drawing Software. https://chemaxon.com/marvin (accessed 2025–12–10).

[31] Siegmund I., Wagner F. (1991). New Method for Detecting Rhamnolipids Excreted by Pseudomonas
Species during Growth on Mineral Agar. Biotechnol.
Technol..

[32] Mulligan N., Catherine, Cooper G., David, Neufeld J., Ronald (2025). Selection of Microbes
Producing Biosurfactants in Media without Hydrocarbons. J. Ferment. Technol..

[33] Jain D. K., Collins-Thompson D. L., Lee H., Trevors J. T. (1991). A Drop-Collapsing
Test for Screening Surfactant-Producing Microorganisms. J. Microbiol. Methods.

[34] Rani M., Weadge J. T., Jabaji S. (2020). Isolation
and Characterization of
Biosurfactant-Producing Bacteria From Oil Well Batteries With Antimicrobial
Activities Against Food-Borne and Plant Pathogens. Front. Microbiol..

[35] Cirigliano M. C., Carman G. M. (1985). Purification and Characterization of Liposan, a Bioemulsifier
from Candida lipolytica. Appl. Environ. Microbiol..

[36] Noll P., Solarte-Toro J. C., Restrepo-Serna D. L., Treinen C., Poveda-Giraldo J. A., Henkel M., Cardona Alzate C. A., Hausmann R. (2024). Limits for Sustainable
Biosurfactant Production: Techno-Economic and Environmental Assessment
of a Rhamnolipid Production Process. Bioresour.
Technol. Rep..

[37] Dextrose Prices, Trends, Chart, News, Index and Demand. https://www.chemanalyst.com/Pricing-data/dextrose-1391 (accessed 2025–12–11).

[38] Rapeseed Oil Prices, Trends, Chart, News, Index and Market Demand. https://www.chemanalyst.com/Pricing-data/rapeseed-oil-1320 (accessed 2025–12–11).

[39] Accepting waste | Wood waste | Puidukäitlus OÜ. Puidukäitlus. https://puidukaitlus.ee/en/services/accepting-waste/ (accessed 2025–12–11).

[40] Wood disposal - Brockmann Krefeld. https://www.brockmann-holz.de/wood-disposal.html (accessed 2025–12–11).

[41] Çakmak H., Güngörmedi G., Dikmen G., Çelik P. A., Çabuk A. (2017). The True Methodology for Rhamnolipid: Various Solvents
Affect Rhamnolipid Characteristics. Eur. J.
Lipid Sci. Technol..

[42] Correia J., Gudiña E. J., Lazar Z., Janek T., Teixeira J. A. (2022). Cost-Effective
Rhamnolipid Production by Burkholderia thailandensis E264 Using Agro-Industrial
Residues. Appl. Microbiol. Biotechnol..

[43] Kumar R., Johnravindar D., Wong J. W. C., Patria R. D., Kaur G. (2023). Economical
Di-Rhamnolipids Biosynthesis by Non-Pathogenic Burkholderia thailandensis
E264 Using Post-Consumption Food Waste in a Biorefinery Approach. Sustainability.

[44] Zhao F., Zhang J., Shi R., Han S., Ma F., Zhang Y. (2015). Production of Biosurfactant by a Pseudomonas aeruginosa Isolate and
its Applicability to in situ Microbial Enhanced Oil Recovery under
Anoxic Conditions. RSC Adv..

[45] Singh P., Tiwary B. N. (2016). Isolation and Characterization
of Glycolipid Biosurfactant
Produced by a Pseudomonas otitidis Strain Isolated from Chirimiri
Coal Mines, India. Bioresour. Bioprocess..

[46] Aranda, F. J. ; Ortiz, A. ; Teruel, J. A. Interaction of Glycolipid Biosurfactants with Model Membranes and Proteins. In Biosurfactants and Sustainability; John Wiley & Sons, Ltd, 2023; pp 195–219.

[47] Dubeau D., Déziel E., Woods D. E., Lépine F. (2009). Burkholderia
thailandensis Harbors Two Identical Rhl Gene Clusters Responsible
for the Biosynthesis of Rhamnolipids. BMC Microbiol..

[48] Déziel E., Lépine F., Dennie D., Boismenu D., Mamer O. A., Villemur R. (1999). Liquid Chromatography/Mass
Spectrometry Analysis of
Mixtures of Rhamnolipids Produced by Pseudomonas aeruginosa Strain
57RP Grown on Mannitol or Naphthalene. BBA -
Mol. Cell Biol. of Lipids.

[49] Joy S., Rahman P. K. S. M., Khare S. K., Sharma S. (2019). Production and Characterization
of Glycolipid Biosurfactant from Achromobacter Sp. (PS1) Isolate Using
One-Factor-at-a-Time (OFAT) Approach with Feasible Utilization of
Ammonia-Soaked Lignocellulosic Pretreated Residues. Bioprocess Biosyst. Eng..

[50] Zhu K., Rock C. O. (2008). RhlA Converts β-Hydroxyacyl-Acyl
Carrier Protein
Intermediates in Fatty Acid Synthesis to the β-Hydroxydecanoyl-β-Hydroxydecanoate
Component of Rhamnolipids in Pseudomonas aeruginosa. J. Bacteriol..

[51] Déziel E., Lépine F., Milot S., Villemur R. (2003). rhlA Is Required for
the Production of a Novel Biosurfactant Promoting Swarming Motility
in Pseudomonas aeruginosa: 3-(3-Hydroxyalkanoyloxy)­Alkanoic Acids
(HAAs), the Precursors of Rhamnolipids. Microbiology.

[52] Ochsner U. A., Fiechter A., Reiser J. (1994). Isolation, Characterization, and
Expression in Escherichia coli of the Pseudomonas aeruginosa rhlAB
Genes Encoding a Rhamnosyltransferase Involved in Rhamnolipid Biosurfactant
Synthesis. J. Biol. Chem..

[53] Rahim R., Ochsner U. A., Olvera C., Graninger M., Messner P., Lam J. S., Soberón-Chávez G. (2001). Cloning and
Functional Characterization of the Pseudomonas aeruginosa rhlC Gene
That Encodes Rhamnosyltransferase 2, an Enzyme Responsible for Di-Rhamnolipid
Biosynthesis. Mol. Microbiol..

[54] Wittgens A., Santiago-Schuebel B., Henkel M., Tiso T., Blank L. M., Hausmann R., Hofmann D., Wilhelm S., Jaeger K.-E., Rosenau F. (2018). Heterologous
Production of Long-Chain Rhamnolipids
from Burkholderia glumae in Pseudomonas putida—a Step Forward
to Tailor-Made Rhamnolipids. Appl. Microbiol.
Biotechnol..

[55] Gudiña E. J., Rodrigues A. I., de Freitas V., Azevedo Z., Teixeira J. A., Rodrigues L. R. (2016). Valorization
of Agro-Industrial Wastes towards the
Production of Rhamnolipids. Bioresour. Technol..

[56] Ramos P., Honda R., Hoek E. M. V., Mahendra S. (2023). Carbon/Nitrogen Ratios
Determine Biofilm Formation and Characteristics in Model Microbial
Cultures. Chemosphere.

[57] Velraeds M. M. C., van der Mei H. C., Reid G., Busscher H. J. (1996). Physicochemical
and Biochemical Characterization of Biosurfactants Released by Lactobacillus
Strains. Colloids Surf., B.

[58] John W. C., Ogbonna I. O., Gberikon G. M., Iheukwumere C. C. (2021). Evaluation
of Biosurfactant Production Potential of Lysinibacillus fusiformis
MK559526 Isolated from Automobile-Mechanic-Workshop Soil. Braz. J. Microbiol..

[59] Colovic M. B., Vasic V. M., Djuric D. M., Krstic D. Z. (2018). Sulphur-Containing
Amino Acids: Protective Role Against Free Radicals and Heavy Metals. Curr. Med. Chem..

[60] Kizil R., Irudayaraj J., Seetharaman K. (2002). Characterization of Irradiated Starches
by Using FT-Raman and FTIR Spectroscopy. J.
Agric. Food Chem..

[61] McAvan B. S., France A. P., Bellina B., Barran P. E., Goodacre R., Doig A. J. (2020). Quantification of
Protein Glycation Using Vibrational
Spectroscopy. Analyst.

[62] Yang S., Zhang Q., Yang H., Shi H., Dong A., Wang L., Yu S. (2022). Progress in Infrared Spectroscopy
as an Efficient Tool for Predicting Protein Secondary Structure. Int. J. Biol. Macromol..

[63] Tatulian, S. A. Structural Characterization of Membrane Proteins and Peptides by FTIR and ATR-FTIR Spectroscopy. In Lipid-Protein Interactions: Methods and Protocols; Kleinschmidt, J. H. , Ed.; Humana Press: Totowa, NJ, 2013; pp 177–218. 10.1007/978-1-62703-275-9_9 23404277

[64] Yang H., Yang S., Kong J., Dong A., Yu S. (2015). Obtaining
Information about Protein Secondary Structures in Aqueous Solution
Using Fourier Transform IR Spectroscopy. Nat.
Protoc..

[65] Gharaie S., Ohadi M., Hassanshahian M., Shakibaie M., Shahriary P., Forootanfar H. (2023). Glycolipopeptide Biosurfactant from
Bacillus pumilus SG: Physicochemical Characterization, Optimization,
Antibiofilm and Antimicrobial Activity Evaluation. 3 Biotech.

[66] Soyuer K., Bilen Ozyurek S. (2024). An Eco-Friendly Approach to Biosurfactant
Production
Using Low-Cost Wastes. J. Dispersion Sci. Tech.

[67] Pemmaraju S. C., Sharma D., Singh N., Panwar R., Cameotra S. S., Pruthi V. (2012). Production of Microbial Surfactants from Oily Sludge-Contaminated
Soil by Bacillus subtilis DSVP23. Appl. Biochem.
Biotechnol..

[68] Lazzem A., Galai H., Landoulsi A., Chatti A., El May A. (2025). Characterization
of a New Glycolipopeptide Biosurfactant Produced by a Chrysene-Degrading
Strain Achromobacter aegrifaciens. Appl. Biochem.
Biotechnol..

[69] Mielke S. P., Krishnan V. V. (2009). Characterization of Protein Secondary
Structure from
NMR Chemical Shifts. Prog. Nucl. Magn. Reson.
Spectrosc..

[70] Wishart D. S., Sykes B. D., Richards F. M. (1992). The Chemical Shift
Index: A Fast
and Simple Method for the Assignment of Protein Secondary Structure
through NMR Spectroscopy. Biochemistry.

[71] Shoulaifar, T. K. Chemical Changes in Biomass during Torrefaction; Åbo Akademi University: Finland, 2016. https://www.doria.fi/handle/10024/118713 (accessed 2025–08–04).

[72] Sultana S., Sultana R., Abdullah Al-Mansur M., Ahedul Akbor M., Akter Bhuiyan N., Ahmed S., Yasmin S., Jamal A. S. I. M. (2024). An
Industrially Potent Rhamnolipid-like Biosurfactant Produced from a
Novel Oil-Degrading Bacterium, Bacillus velezensis S2. RSC Adv..

[73] Khademolhosseini R., Jafari A., Mohammad Mousavi S., Hajfarajollah H., Akbari Noghabi K., Manteghian M. (2019). Physicochemical Characterization
and Optimization of Glycolipid Biosurfactant Production by a Native
Strain of Pseudomonas aeruginosa HAK01 and Its Performance Evaluation
for the MEOR Process. RSC Adv..

[74] Barbosa F. G., Marcelino P. R. F., Lacerda T. M., Philippini R. R., Giancaterino E. T., Mancebo M. C., dos Santos J. C., da Silva S. S. (2022). Production, Physicochemical
and Structural Characterization
of a Bioemulsifier Produced in a Culture Medium Composed of Sugarcane
Bagasse Hemicellulosic Hydrolysate and Soybean Oil in the Context
of Biorefineries. Fermentation.

[75] Simões C. R., da Silva M. W. P., de
Souza R. F. M., Hacha R. R., Merma A. G., Torem M. L., Silvas F. P. C. (2024). Biosurfactants: An Overview of Their
Properties, Production, and Application in Mineral Flotation. Resources.

[76] Cavalero D. A., Cooper D. G. (2003). The Effect of Medium Composition
on the Structure and
Physical State of Sophorolipids Produced by Candida bombicola ATCC
22214. J. Biotechnol..

[77] Jain R. M., Mody K., Joshi N., Mishra A., Jha B. (2013). Production
and Structural Characterization of Biosurfactant Produced by an Alkaliphilic
Bacterium, Klebsiella Sp.: Evaluation of Different Carbon Sources. Colloids Surf., B.

[78] Bellavita R., Braccia S., Galdiero S., Falanga A. (2023). Glycosylation and Lipidation
Strategies: Approaches for Improving Antimicrobial Peptide Efficacy. Pharmaceuticals.

[79] Rather M. A., Bharadwaj R., Haldar M., Sengar D. S., Verma H. G., Goswami P. K., Mandal M. (2025). Biodegradation of Crude Oil by Biosurfactant-Producing
Microaerophilic Bacterium Pseudomonas aeruginosa MAR1. Sci. Total Environ..

[80] Bouchemal K., Briançon S., Perrier E., Fessi H. (2004). Nano-Emulsion Formulation
Using Spontaneous Emulsification: Solvent, Oil and Surfactant Optimisation. Int. J. Pharm..

[81] Chu Y., Zhao K., Gao S., Wang W., Liu J., Zhang J., Pu W., Liu R. (2024). Experimental Study
on Self–Emulsification of Shale Crude Oil by Natural Emulsifiers. J. Dispersion Sci. Technol..

[82] Al-Sakkaf M. K., Onaizi S. A. (2024). Effects of Emulsification
Factors on the Characteristics
of Crude Oil Emulsions Stabilized by Chemical and Biosurfactants:
A Review. Fuel.

[83] Liu J., Li Y., Lun Z., Zhang Y., Yang P., Tang X., Zhang Q. (2024). Factors, Mechanisms,
and Kinetics of Spontaneous Emulsification for
Heavy Oil-in-Water Emulsions. Molecules.

[84] Bognolo G. (1999). Biosurfactants
as Emulsifying Agents for Hydrocarbons. Colloids
Surf. A Physicochem. Eng. Asp..

[85] Stamatelatou, K. ; Pakou, C. ; Lyberatos, G. Occurrence, Toxicity, and Biodegradation of Selected Emerging Priority Pollutants in Municipal Sewage Sludge. In Comprehensive Biotechnology Vol. 6: Environmental Biotechnology and Safety; Pergamon Press: Oxford, United Kingdom, 2011; pp 473–484.

[86] Rhamnolipids from Pseudomonas aeruginosa 90 Sigma-Aldrich. https://www.sigmaaldrich.com/EE/en/product/sigma/r90?srsltid=AfmBOoqw3JqJBEeKx3mMe6XafEbpCI5tahGM33WXoCMpRj20p25i0ax- (accessed 2025–11–28).

